# Dynamic Relocation of Copper Catalysts in Gas Diffusion Electrodes during CO_2_ Electroreduction

**DOI:** 10.1021/jacs.5c07944

**Published:** 2025-06-25

**Authors:** Daiko Takamatsu, Naoto Fukatani, Akio Yoneyama, Tatsumi Hirano, Kakuro Hirai, Shin Yabuuchi, Koichi Watanabe, Kazuhide Kamiya, Shuji Nakanishi

**Affiliations:** † Center for Exploratory Research, 13480 Research & Development Group, Hitachi, Ltd., 2520, Akanuma, Hatoyama-machi, Saitama 350-0395, Japan; ‡ Research Center for Solar Energy Chemistry, Graduate School of Engineering Science, 13013 Osaka University, 1-3, Machikaneyama, Toyonaka, Osaka 560-8531, Japan; § Innovative Catalysis Science Division, Institute for Open and Transdisciplinary Research Initiatives (ICS-OTRI), 2-1, Yamada-oka, Suita, Osaka 565-0871, Japan

## Abstract

Developing technologies that convert CO_2_ into valuable carbon products using renewable energy is of growing significance. Copper (Cu) is a unique electrocatalyst capable of reducing CO_2_ to value-added multicarbon (C_2+_) compounds. While recent *in situ* studies have elucidated the dynamic evolution of Cu catalysts during electrochemical CO_2_ reduction reactions (CO_2_RR), the relationship between catalyst behavior in gas diffusion electrodes (GDEs) and C_2+_ product selectivity at industrially relevant current densities remains insufficiently understood. In this study, we examined the correlation between the structure, chemical state, and C_2+_ selectivity of Cu catalysts in Cu-GDEs during CO_2_RR operation at current densities exceeding 200 mA/cm^2^. *Ex situ* and *in situ* scanning X-ray fluorescence microscopy revealed significant relocation of Cu within the GDE after CO_2_RR. *In situ* X-ray absorption spectroscopy identified the presence of Cu^1+^ species during operation, indicating that Cu relocation proceeds via a dissolution-redeposition. The dissolution-redeposition behavior was found to be pH-dependent and more pronounced at high pH. Online gas chromatography demonstrated that the decrease in C_2+_ selectivity over time was primarily due to flooding, overshadowing the impact of Cu relocation on C_2+_ selectivity. These findings provide important insights for designing stable and highly selective Cu-based GDEs for practical CO_2_ electrolyzers.

## Graphical abstract

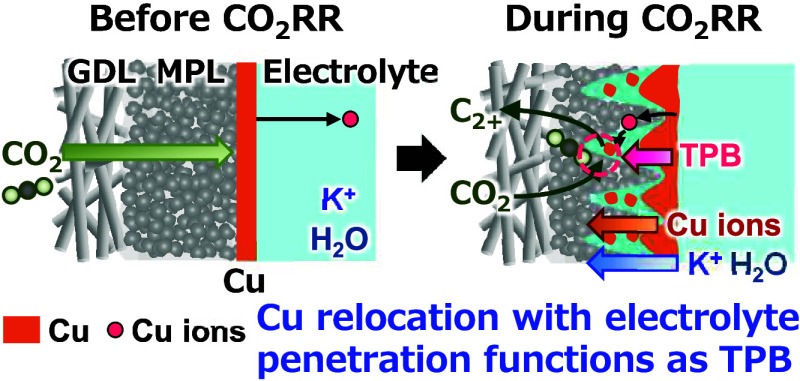

## 1 Introduction

The conversion of CO_2_ into valuable chemicals and fuels through electrochemical CO_2_ reduction reactions (CO_2_RR) using renewable energy sources offers a promising path toward a sustainable, carbon-neutral society.^1–5^ Among the various CO_2_ reduction products, multicarbon (C_2+_) compounds such as ethylene and ethanol have attracted considerable attention due to their higher energy density and economic value.^6,7^ Copper (Cu) has been extensively investigated as an electrocatalyst for CO_2_RR due to its unique ability to facilitate the formation of C_2+_ products.^2,3,7,8^ Nevertheless, the efficiency, selectivity, and reaction rates of Cu-based catalysts remain insufficient for practical implementation. To achieve the economic feasibility required for industrial applications, CO_2_ electrolyzers must operate at current densities exceeding 200 mA/cm^2^.^9,10^ This has been made possible using gas diffusion electrodes (GDEs), in which catalysts are deposited on gas diffusion layers (GDLs). GDEs overcome the mass transport limitation of CO_2_ in aqueous solutions by supplying CO_2_ directly to the catalyst in gaseous form, thereby enhancing reaction rates at high current densities.^11–15^ For developing commercially relevant CO_2_ electrolyzers using GDEs, a comprehensive understanding of the catalyst structure and the local reaction environment under practical operating conditions is essential. In this context, *in situ* characterization techniques are expected to be critical in elucidating catalyst behavior and guiding rational electrode design.

Recent *in situ* characterization techniques, such as transmission electron microscopy (TEM),^16,17^ scanning probe microscopy (SPM),^18–20^ X-ray absorption spectroscopy (XAS),^21–23^ infrared spectroscopy (IR),^24,25^ Raman spectroscopy,^26,27^ and their complementary analyses,^28–30^ have shown that Cu catalysts undergo significant changes in chemical state, structure, and interfacial properties during CO_2_RR. However, most of these *in situ* studies have been conducted using batch cell configurations rather than GDE-type cells. In batch cells, the low solubility of CO_2_ in aqueous electrolytes (typically CO_2_-saturated KHCO_3_ solutions) severely limits the achievable CO_2_ reduction current density, often restricting it to below a few tens of mA/cm^2^. This constraint hinders the investigation of catalytic behavior under industrially relevant conditions. In contrast, practical CO_2_ electrolyzers using GDEs, such as flow cells and membrane electrode assembly (MEA) cells, operate at much higher current densities. Despite their importance, the structural evolution and chemical dynamics of Cu catalysts in GDEs under such conditions remain poorly understood. This lack of knowledge makes it difficult to fully elucidate the formation mechanisms of C_2+_ products and hinders the rational design of high-performance CO_2_RR systems.

This study aims to elucidate the structure and chemical state of Cu catalysts within Cu-GDEs during CO_2_RR at practical current densities, as well as the influence of electrolyte penetration, which can lead to flooding. First, the Faradaic efficiency (FE) of each CO_2_RR product is quantified in a neutral electrolyte (1 M KCl as catholyte) using a flow cell system with Cu-GDE as cathode. The spatial distributions of Cu catalysts and K (as an indicator of electrolyte penetration) in the Cu-GDE cross-section before and after CO_2_RR are systematically analyzed by *ex situ* scanning X-ray fluorescence microscopy (SXFM) and X-ray computed tomography (CT). Furthermore, the temporal changes in the chemical state of the Cu catalysts during electrolysis at practical current densities are studied by *in situ* XAS. Subsequently, the time-dependent variations in the FE of each gaseous product were investigated under continuous constant current operation in neutral (1 M KCl) and alkaline (1 M KOH) catholytes. Finally, the implications of Cu relocation on C_2+_ product selectivity and flooding are discussed based on *in situ* SXFM observations of Cu structural changes during CO_2_RR.

## 2 Results and Discussion

### 2.1 Morphological Changes of Cu-GDEs during Electrolysis in Neutral Electrolyte under CO_2_ or Ar Supply

Cu­(*x*)-GDEs (thickness of Cu: *x* nm) were prepared by magnetron sputtering of Cu onto a commercial carbon-based gas diffusion layer (GDL) with a microporous layer (MPL) (Figure S1). The typical thicknesses of the MPL and GDL were approximately 70 and 100 μm, respectively (Figure S2). CO_2_RR experiments were conducted in a three-chamber flow cell system employing a Cu(300)-GDE as the working electrode, platinum (Pt) mesh as the counter electrode, and Ag/AgCl as the reference electrode (Figure S3).^15^ The FEs of CO_2_RR products were determined by continuous operation of chronopotentiometry (CP) for 20 min in a neutral electrolyte (1 M KCl) serving as the catholyte. Figure 1a presents the CO_2_RR product distributions obtained for varying total current densities (*J*_total_) in the Cu(300)-GDE system (see Supporting Note S1 for details on FE calculations). The FE for C_2+_ products (FE_C2+_), comprising C_2_H_4_, C_2_H_5_OH, CH_3_COOH, and C_3_H_7_OH, was found to be approximately 80% across a broad range of current densities (−400 to −800 mA/cm^2^). The maximum FE_C2+_ reached 82% at −400 mA/cm^2^, with FE_C2H4_ = 41% and FE_C2H5OH_ = 36%. Given that the partial current density of C_2+_ (*J*_C2+_) exceeds −300 mA/cm^2^ (Figure S4), the catalytic behavior of CO_2_RR under practical current densities can be effectively analyzed. However, upon increasing the current density to −1000 mA/cm^2^, a decline in FE_C2+_ and a corresponding increase in FE_H2_ was observed. The reduction in FE_C2+_ at elevated current densities is attributable to flooding, as discussed in subsequent sections. Figure 1b illustrates *ex situ* scanning electron microscopy (SEM) and energy dispersive X-ray spectroscopy (EDX) images of Cu(300)-GDE cross sections before (pristine) and after CO_2_RR. The pristine Cu(300)-GDE exhibited a uniformly deposited Cu catalyst layer (CL) on the MPL surface. In contrast, following CP at −400 mA/cm^2^ for 20 min under CO_2_ supply, Cu catalysts were observed not only near the MPL surface but also within the MPL. Notably, after CP at −800 mA/cm^2^ for 20 min under CO_2_ supply, Cu was no longer observed near the MPL surface but instead diffused further into the MPL. These SEM-EDX results indicate that the Cu-CL morphology undergoes significant structural transformations during CO_2_RR.

**Figure 1:**
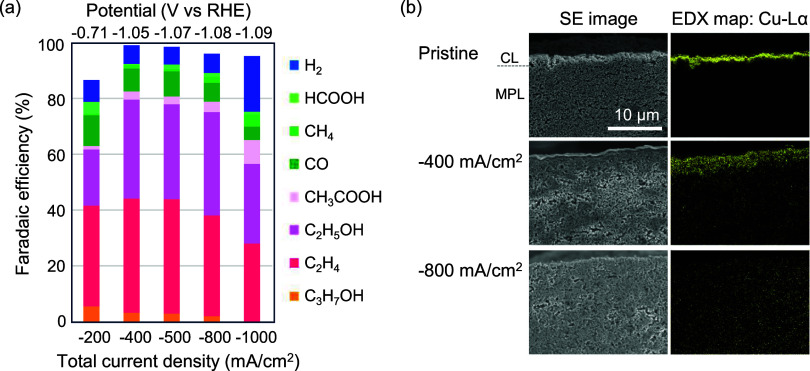
(a) Current density dependence of FEs of CO_2_RR products with Cu(300)-GDE as the cathode in neutral electrolyte (1 M KCl as catholyte). The operation time for each CP was fixed at 20 min. (b) SEM-EDX images (×5000) of Cu(300)-GDE cross-sectional samples: as-prepared (pristine), after CP at −400 mA/cm^2^ for 20 min and after CP at −800 mA/cm^2^ for 20 min. SE image: secondary electron image.

Figure 2a presents *ex situ* SXFM images of Cu–Kα (Cu map) and K–Kα (K map) for the same samples shown in Figure 1b. See Figure S5 for details of the *ex situ* SXFM measurement system. It should be noted that the Cu distribution width observed in the SXFM image of the pristine sample (Figure 2a) appears broader than in the corresponding SEM-EDX image (Figure 1b). This discrepancy arises due to differences in detection depth: X-ray fluorescence analysis has a significantly greater detection depth compared to SEM (Figure S6c). Additionally, the GDE surface exhibits irregularities on the scale of tens of microns (see X-ray CT images in Figure S6b). Consequently, a Cu film with uniform submicron thickness may appear wider in SXFM due to the surface topography of the GDE, as illustrated in Figure S6d. Cu maps of postelectrolysis samples (CP at −400 and −800 mA/cm^2^ for 20 min under CO_2_ supply) show that Cu was distributed within the MPL, whereas the pristine sample remains Cu on the MPL surface (Figure 2a). Furthermore, in both postelectrolysis samples, K distribution in the K map closely resembles the Cu distribution in the Cu map (Figure 2a). Intensity profiles of Cu and K along the *z*-axis (Figure 2c) indicate that the penetration depths of both elements into the MPL increase with increasing charge passed. The splitting observed in Cu and K intensity profiles after CP at −800 mA/cm^2^ (denoted by red arrows in Figure 2c) suggests that Cu is concentrated at the apical electrolyte region, implying that CO_2_RR reaction sites dynamically migrate toward the electrolyte interface along with dissolved Cu. Figure 2b displays *ex situ* SXFM images of Cu(300)-GDE subjected to CP at −400 mA/cm^2^ for 20 min under argon (Ar) supply. Under Ar supply, Cu remained localized on the MPL surface, whereas K penetrated deeper into the MPL. Comparative analysis of Cu and K intensity profiles for Cu(300)-GDE operated under CO_2_ and Ar supply is shown in Figure 2d. Under Ar supply, Cu does not relocate within the MPL, while under CO_2_ supply, Cu relocation into the MPL occurs. *Ex situ* spectral X-CT further confirms that Cu relocation occurs exclusively under CO_2_ supply (Supporting Note S2 and Figure S7). The deeper penetration of K into the MPL observed under Ar supply (Figure 2d) indicates that electrolyte flooding progresses more rapidly under Ar supply than under CO_2_ supply despite the identical electrolysis charges. The relatively negative potential observed under Ar supply at the same current (Figure 3b) suggests that electrowetting effects of the carbon MPL are more pronounced under Ar supply, consistent with previous findings by Yang et al.^31^ Additionally, the dynamic Cu relocation observed in the Cu(300)-GDE cross-section during electrolysis under CO_2_ supply in a neutral electrolyte was also confirmed by *in situ* SXFM (Supporting Note S3 and Figures S8–S12).

**Figure 2:**
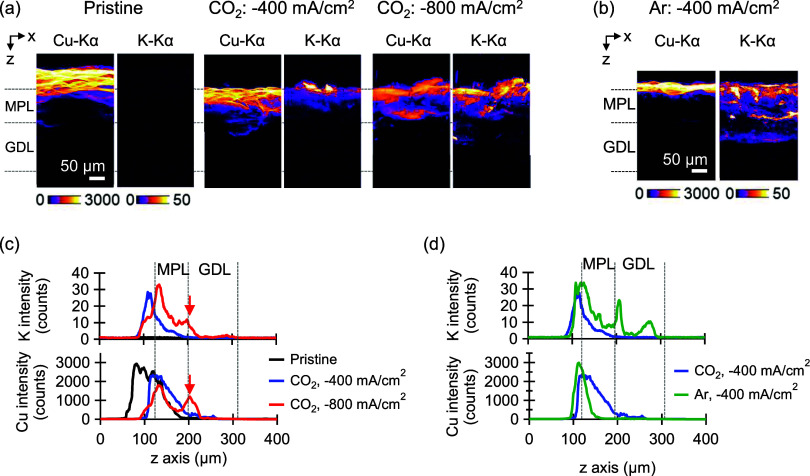
*Ex situ* SXFM images of Cu(300)-GDE cross-section. (a) Cu and K maps of pristine, after CP at −400 mA/cm^2^ for 20 min and after CP at −800 mA/cm^2^ for 20 min under CO_2_ supply in neutral catholyte (1 M KCl). (b) Cu and K maps of after CP at −400 mA/cm^2^ for 20 min under Ar supply in neutral catholyte (1 M KCl). (c) Intensity profiles of Cu and K along *z*-axis averaged on *x*-axis of (a). (d) Intensity profiles of Cu and K along *z*-axis averaged on *x*-axis of (b). The results of CP at −400 mA/cm^2^ under CO_2_ supply are also shown for comparison.

**Figure 3:**
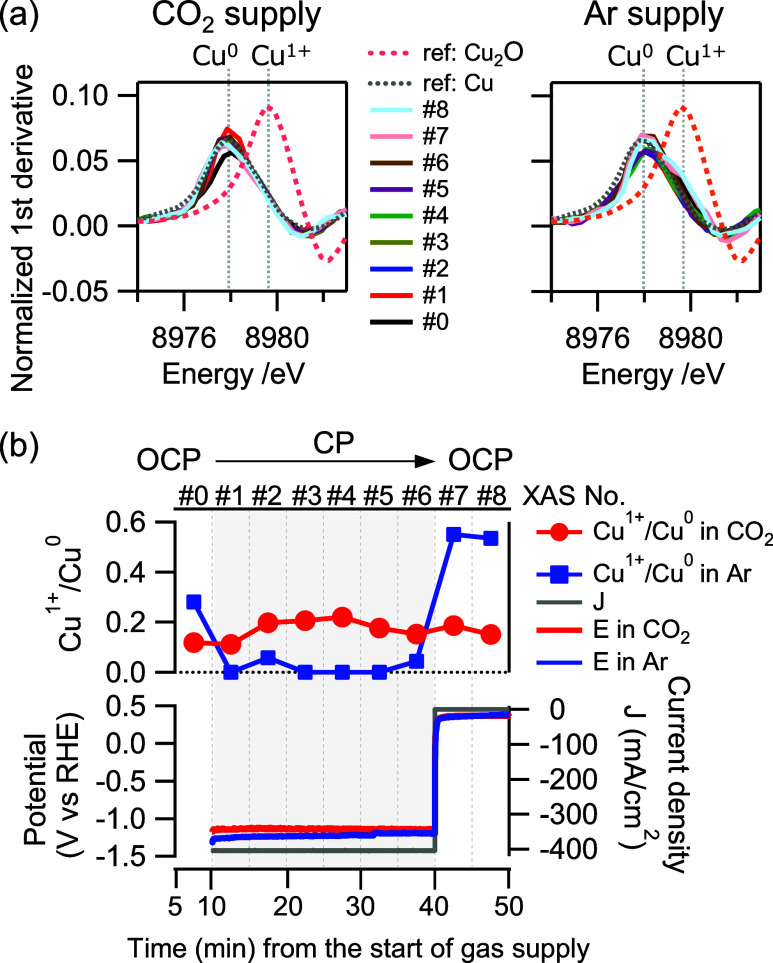
(a) *In situ* normalized Cu–K edge XANES first derivative spectra of the Cu(70)-GDE during CP at −400 mA/cm^2^ and OCP under CO_2_ or Ar supply in neutral electrolyte (1 M KCl), together with the standard samples. (b) Plot of [integrated intensity of Cu^1+^]/[integrated intensity of Cu^0^] calculated from multipeak-fitting of (a) and corresponding CP-OCP curves. The horizonal axis is the time elapsed from the start of gas supply to the cell.

### 2.2 Chemical States of Cu during Electrolysis in Neutral Electrolyte under CO_2_ or Ar Supply

The above-mentioned *ex situ* and *in situ* SXFM results indicate that Cu migration into the MPL during CO_2_RR is closely linked to the chemical state of Cu, including its dissolved ionic form (Cu^+^). To further investigate the chemical state of Cu under electrolysis conditions, *in situ* Cu–K edge XAS measurements were conducted using the flow cell modified to allow X-ray measurements (Figure S13). It should be noted that the Cu–K fluorescence X-ray signal represents the average XAS spectrum of all Cu present within the X-ray irradiation area. Due to the predominant presence of Cu^0^ in Cu(300)-GDE, where Cu^+^ species were buried and could not be directly quantified, *in situ* XAS measurements were instead conducted on a Cu(70)-GDE system, which features a thinner CL thickness to facilitate the detection of Cu^+^ embedded within the bulk Cu^0^ structure. Figure 3a presents the *in situ* normalized first derivative spectra of Cu–K edge X-ray absorption near edge structure (XANES) for Cu(70)-GDE during constant current electrolysis (CP at −400 mA/cm^2^, approximately −1.1 V_RHE_) and at open circuit potential (OCP, approximately +0.4 V_RHE_) under CO_2_ and Ar supply, along with reference standard samples. While the overall spectral profiles of the first derivative XANES remain similar, a distinct variation in peak intensity at the Cu^1+^ energy position is observed between CO_2_ and Ar supply conditions (Figure 3a). A quantitative methodology for determining the ratio of Cu^0^ and Cu^1+^ from first derivative XANES spectra, as previously reported,^21^ was employed for this analysis. The complete XANES data set, including the multiple peak fitting of the first derivative XANES spectra based on characteristic energy positions of Cu^0^ and Cu^1+^, is provided in Figures S14 and S15. The Cu^1+^/Cu^0^ ratio, defined as the integrated intensity of Cu^1+^ relative to Cu^0^, is plotted in Figure 3b. During CP at −400 mA/cm^2^ under CO_2_ supply conditions, the Cu^1+^/Cu^0^ ratio remained approximately 0.2 (Figure 3b, red), while it was nearly 0 under Ar supply (Figure 3b, blue). This suggests that a persistent concentration of Cu^1+^ species was maintained under CO_2_ supply despite cathodic current application, whereas under Ar supply, Cu was predominantly present as Cu^0^ with no detectable Cu^1+^ species. Furthermore, under the experimental conditions of *in situ* XAS, Cu^2+^ was not observed during electrolysis, regardless of CO_2_ or Ar supply.

The horizontal axis in Figure 3b represents the time elapsed from the start of CO_2_ or Ar gas supply to the cell. Notably, approximately 10 min transpired before the current was applied. In the case of CO_2_ supply, the pH of the KCl catholyte (initial pH ∼ 7) decreased to 5.5 within this period, indicating that the initial XANES measurement at OCP (#0) was conducted under mildly acidic conditions. According to the Pourbaix diagram,^32^ Cu dissolution is influenced by both acidic and highly alkaline environments: Cu near OCP (0 V_SHE_) exists as Cu^0^(s) or Cu_2_O(s) at neutral pH, as Cu^+^ at lower pH, and as Cu­(OH)_2_^–^ at higher pH.^32–35^ The substantial increase in the Cu^1+^/Cu^0^ ratio at OCP under Ar supply (Figure 3b, blue) suggests that Cu underwent oxidation to Cu_2_O. In contrast, under CO_2_ supply, the Cu^1+^/Cu^0^ ratio remained stable regardless of cathodic current application, indicating the continuous presence of Cu^1+^ species throughout electrolysis (Figure 3b, red). The existence of Cu^1+^ under CO_2_RR conditions has also been reported in prior studies on Cu-based catalysts.^36–38^ However, the presence of Cu^1+^ in CO_2_RR conditions contradicts predictions based on the Pourbaix diagram, implying that reaction kinetics and local microenvironmental effects play a significant role in stabilizing thermodynamically unfavorable phases during CO_2_RR. Vavra et al. proposed that adsorbed *CO intermediates form Cu-adsorbate complexes, which can persist in solution under CO_2_RR operating conditions.^39^ We speculate that dissolved Cu^1+^ interacts with CO_2_ to form a stable complex ion, potentially [Cu-*CO]^+^, which maintains a detectable concentration within the MPL throughout CO_2_RR. To evaluate whether carbonate ions or CO-derived anions play a role in Cu migration, the Cu distribution within Cu(300)-GDE postelectrolysis in KCl and KHCO_3_ catholytes was examined via scanning transmission electron microscopy (STEM)-EDX (Figure S16). Under CO_2_ supply, Cu relocation was observed regardless of whether KCl or KHCO_3_ was used as the electrolyte (Figure S16a,b). In contrast, under Ar supply, Cu relocation was observed in KHCO_3_ but absent in KCl (Figure S16c,d). These results suggest that the presence of carbonate ions is essential for facilitating Cu migration.

### 2.3 Cu Solubility and CO_2_RR Selectivity as a Function of Initial Electrolyte pH

Regardless of the gas type (CO_2_ or Ar) supplied to the flow cell depicted in Figure S3, the presence of dissolved Cu species in the catholyte after OCP and CP was confirmed by inductively coupled plasma mass spectroscopy (ICP-MS) (Table S1). The concentration of dissolved Cu in the KCl catholyte (initial pH ∼ 7) after 30 min of OCP corresponded to approximately 15% of the initial Cu(300)-GDE loading, yielding a dissolution rate of ∼ 0.5%/min. This dissolution rate aligns with findings reported by Kok et al., who observed Cu dissolution under OCP conditions using Cu-PTFE GDE cathodes in a neutral catholyte (1 M KHCO_3_, pH = 8.3),^40^ further corroborating the dependence of Cu dissolution on the initial catholyte pH. Notably, the amount of dissolved Cu in the KOH catholyte (initial pH ∼ 14) after 30 min of OCP exceeded that in KCl catholyte by more than 3-fold (Table S1). The migration of dissolved Cu complexes is enhanced under locally alkaline conditions, where the presence of OH^–^ ions induces anomalous oxidation-redeposition cycles of Cu.^41,42^ Since Cu under OCP is in a relatively high oxidation state, the formation of Cu–OH complexes may be promoted at elevated pH levels, potentially increasing dissolution regardless of the supplied gas species (CO_2_ or Ar). In contrast, the concentration of dissolved Cu in the catholyte following CP electrolysis was lower than that observed under OCP conditions (Table S1). This decrease in dissolved Cu levels can be attributed to the processes where Cu species transported into the MPL along with the catholyte are redeposited within the MPL during CP, minimizing Cu dissolution into the bulk electrolyte. These results suggest that the pH and composition of the electrolyte influence Cu dissolution and redeposition mechanisms.

To evaluate the dependence of C_2+_ selectivity on the initial electrolyte pH, time-resolved FEs of gaseous products were monitored via online gas chromatography (GC) during continuous CP electrolysis at −400 mA/cm^2^ (Figure 4). FE_C2H4_ was employed as an indicator of sustained C_2+_ selectivity. In the neutral catholyte (1 M KCl, initial pH ∼ 7), FE_C2H4_ was initially high at 39% and remained above 30% for approximately 90 min (Figure 4a). Subsequently, FE_C2H4_ declined, reaching 0% after 160 min. The maximum FE_C2H4_ observed (∼ 40%) is consistent with typical values reported for Cu-based GDE, and the trend of decreasing FE_C2H4_ coupled with increasing FE_H2_ over extended operation is in agreement with previous studies.^40^ Electrolysis was halted upon reaching 0% FE_C2H4_, after which the cell was disassembled, and the GDE was subjected to solvent-based washing and drying to remove accumulated water and salts (denoted by red arrows of “washing” in Figure 4). Following reassembly with fresh electrolyte, CP electrolysis resumed, and FE_C2H4_ partially recovered to 28%. A similar trend was observed in the alkaline catholyte (1 M KOH, initial pH ∼ 14): the initial FE_C2H4_ was 41%, decreased to 0% after 160 min of continuous CP, and recovered to 40% upon washing (Figure 4b). The bulk pH of the KCl catholyte measured at 40 min intervals revealed an increase over operating time (Figure 4a, top). At practical current densities (≥200 mA/cm^2^), the local pH at the reaction interface of neutral catholyte rises, resembling alkaline catholyte conditions.^15,43^ Given that CO_2_RR in Figure 4 was performed at 400 mA/cm^2^, comparable local pH conditions and analogous product selectivity were achieved in both neutral and alkaline systems. Figure 4 also demonstrates that the onset of FE_H2_ increase occurred earlier in KOH (∼ 80 min) than in KCl (∼ 100 min), a phenomenon further clarified in Figure S17. Carbonate formation is promoted wherever dissolved CO_2_ and OH^–^ ions coexist and is further enhanced under increasing alkalinity. Consequently, alkaline conditions expedite salt accumulation within the GDE, which may contribute to operational flooding.^44^ These results indicate that the decrease in C_2+_ selectivity over operating time is primarily due to flooding effects, where salt accumulation impedes gas diffusion within the GDE, restricts CO_2_ supply, and promotes the hydrogen evolution reaction (HER).

**Figure 4:**
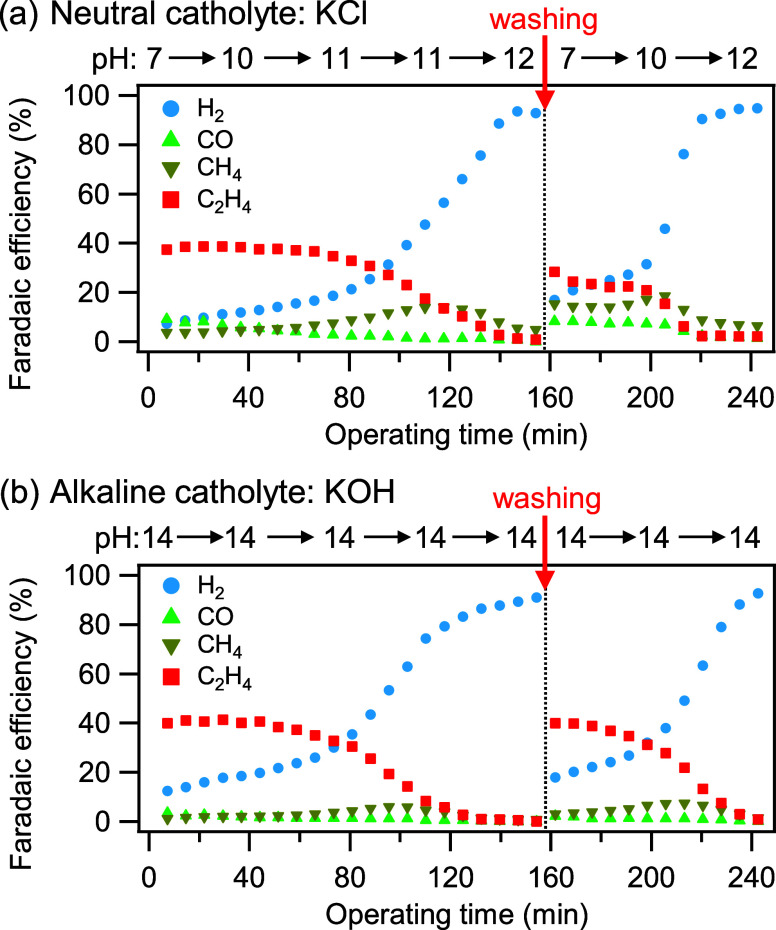
FE curves of gas products (H_2_, CO, CH_4_, and C_2_H_4_) as a function of electrolysis time at −400 mA/cm^2^ under CO_2_ supply. A Cu(300)-GDE was used as cathode, with (a) neutral catholyte (1 M KCl) and (b) alkaline catholyte (1 M KOH). Washing of the cathode was examined at the indicated red arrow position, where the GDE was removed, solvent-washed, and dried before being reused to reassemble the cell with flesh electrolyte. The bulk catholyte pH values recorded every 40 min are shown at the top.

### 2.4 Effect of Cu Relocation on C_2+_ Selectivity and CO_2_RR Stability

Although significant Cu relocation within the GDE was observed within 20 min of CP in 1 M KCl catholyte (Figures 2, S7 and S11), FE_C2H4_ remained consistently high both before and after 20 min (Figure 4a). These results indicate that Cu relocation does not directly govern C_2+_ selectivity. To further investigate the effect of Cu relocation on electrochemically active sites, we measured the electrochemically active surface area (ECSA), a proxy for the electrolyte-wetted electrode surface.^12,44^ An efficient triple-phase boundary (TPB; gas–liquid–solid interface) prevents excessive electrode wetting and results in a relatively low electrochemical double layer capacitance (*C*_dl_).^44^ Figure S20 presents the *C*_dl_ values of Cu-GDE in 1 M KCl and 1 M KOH before and after 10 min of CP at −400 mA/cm^2^, derived from cyclic voltammetry (CV) measurements in 1 M KCl (Figure S18) and 1M KOH (Figure S19). In both cases, C_dl_ was initially low but increased after CP, indicating progressive electrode wetting. Notably, washing the GDE reduced *C*_dl_ to its initial or lower values (Figure S20c), with most C_2+_ selectivity restored (Figure 4). These results suggest that the TPB can be reconstructed by removing accumulated salts, even after Cu relocation. Initially, we anticipated that Cu relocation would have a significant effect on C_2+_ selectivity. However, these findings reveal that the decline in C_2+_ selectivity over time is primarily attributed to flooding rather than Cu relocation. However, this is likely because the dominant effect of flooding masks the influence of Cu relocation on C_2+_ selectivity. Under conditions where flooding is effectively suppressed, Cu relocation may still exert a significant influence on C_2+_ selectivity. In such cases, the formation of highly dispersed Cu nanoparticles via dissolution and redeposition could further enhance C_2+_ selectivity.^45^ Nevertheless, if Cu relocation progresses further and Cu catalyst is completely lost from the GDE via dissolution, the electrode would become a simple carbon electrode, resulting in irreversible loss of CO_2_RR activity.

Figure 5 schematically illustrates the Cu relocation process within Cu-GDE cathodes during CO_2_RR. Regardless of electrolyte pH, Cu dissolves into electrolyte under OCP conditions (Figure 5a). Cu dissolution is more pronounced in alkaline electrolytes (Table S1); therefore, minimizing time spent at OCP is critical for ensuring catalyst longevity, irrespective of electrolyte pH. Upon cathodic current application, Cu dissolves as Cu complex ions and undergoes dynamic migration within the GDE through cyclic dissolution and redeposition, ultimately resulting in Cu relocation (Figure 5b). This Cu relocation process is more pronounced in alkaline electrolytes and was confirmed by *in situ* SXFM analysis of Cu distribution during CP-OCP cycles under CO_2_ supply (Figure S21). Indeed, the spatial distribution of Cu maps after CP-OCP cycles reveal more extensive Cu dispersion within the MPL in alkaline electrolytes than in neutral electrolyte (Figure S22). We speculate that the pronounced Cu relocation observed in alkaline conditions may result from cathodic corrosion under highly reducing potentials.^28,46^

**Figure 5:**
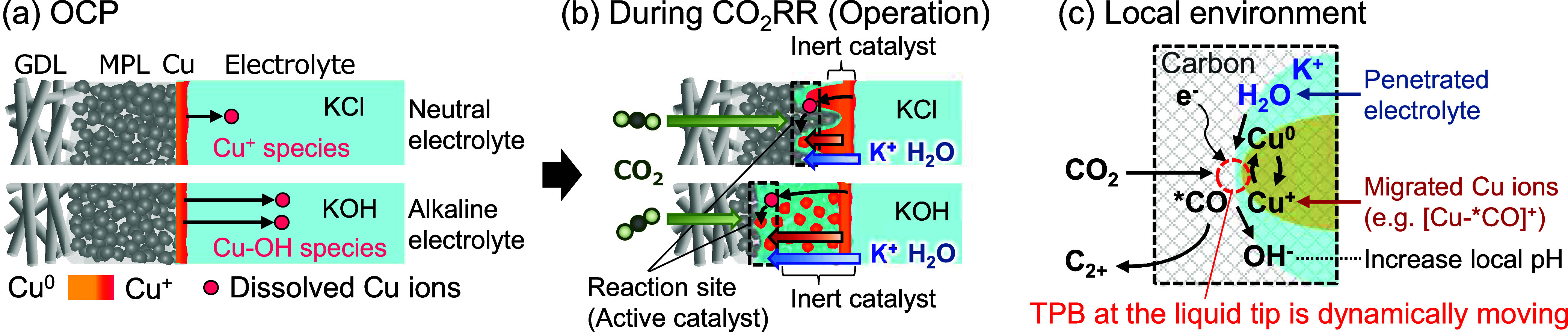
Schematic depiction of the Cu relocation process. (a) Cu dissolves in the electrolyte at OCP, with dissolution being more pronounced in an alkaline catholyte (e.g., KOH) than in a neutral catholyte (e.g., KCl). (b) The reaction site shifts as electrolyte penetration progresses. Cu ions migrate into the MPL/GDL and relocate during CO_2_RR operation. The extent of liquid penetration (Cu migration) is greater in an alkaline electrolyte than in a neutral electrolyte. (c) A magnified view of the local environment at the reaction site. CO_2_RR proceeds at the triple-phase boundary (TPB) at the electrolyte penetration tip, accompanied by the reposition of dissolved Cu species.

Previous studies have highlighted the importance of residual Cu^+^ species in CO_2_RR for improving C_2+_ selectivity.^36–38^ Additionally, nanoparticle catalyst morphology has been reported to influence C–C bond formation in CO_2_RR.^45^ Although Cu morphology and chemical state play a role in determining CO_2_RR performance,^42^ their effect is likely substantial only under nonflooded conditions. We propose that the efficient formation of TPB within the local reaction environment is more critical for achieving high C_2+_ selectivity in commercially viable CO_2_RR systems utilizing GDEs. Traditionally, CO_2_RR in GDEs has been conceptualized as a TPB-based mechanism, where catalyst particles remain stationary at the GDE while the gas–liquid interface dynamically moves in within the GDE.^47,48^ Recently, the double-phase boundary (DPB; liquid–solid interface) has been suggested as an alternative model, treating CO_2_ in GDEs as dissolved CO_2_.^49^ Our findings present a novel perspective: Cu catalyst dynamically migrates within GDEs along with the electrolyte and function as a dynamic TPB within the GDE interior (Figure 5c). This interpretation aligns with prior reports suggesting that the hydrophobic microenvironment within GDEs enhances TPB efficiency and improves CO_2_RR selectivity.^12,50–52^ Moreover, recent operando X-ray scattering investigations of GDE-type CO_2_RR cells have underscored the detrimental effects of salt precipitation in the GDE cathode under high current densities (≥100 mA/cm^2^), leading to decreased CO_2_RR selectivity.^53–55^ Our results demonstrate that C_2+_ selectivity can be partially restored via salt removal through washing; however, once flooding occurs, complete recovery of initial peak performance is challenging (Figures 4 and S17). This suggests that deeply embedded salt precipitates may be difficult to remove by the current washing methods, or that salt deposition may irreversibly damage the porosity of the GDE, thereby increasing permeability and reducing hydrophobicity.^44^ Periodic restoration of GDEs via *ex situ* and *in situ* washing protocols are necessary to prolong CO_2_RR selectivity.^56,57^ As the strategies to mitigate flooding, developing high-activity catalysts with low onset potentials, or the modification and/or substitution of GDL materials to optimize wettability has also been proposed.^31^ Ultimately, technical solutions are required to minimize flooding and improve performance in GDE-type CO_2_RR electrolyzers operated under high current densities.

## 3 Conclusions

This study systematically investigated the correlation between the Cu relocation process and C_2+_ selectivity of Cu-GDE cathodes in CO_2_RR operated at practical current densities. *Ex situ* and *in situ* SXFM results showed that the Cu catalyst undergoes dynamic structural changes during CO_2_RR, migrating and dispersing within the GDE along with electrolyte penetration. *In situ* XAS results showed that Cu migration to the MPL is governed by the dissolution and redeposition of Cu^1+^ species. These Cu^1+^ species may exist as stable dissolved ions, such as [Cu-CO]^+^ complex ions, in the presence of CO_2_ or carbonate ions. Based on these findings, we proposed a mechanism in which the Cu catalyst undergoes continuous relocation within the GDE in conjunction with electrolyte permeation in the CO_2_RR. We also investigated the time-dependent changes in Cu solubility and C_2+_ selectivity as a function of electrolyte pH. It was found that Cu dissolution occurs under OCP conditions, and that the concentration of dissolved Cu increases at higher electrolyte pH. Analysis of the time-dependent variations in the FEs of gaseous products measured by online GC during continuous CO_2_RR revealed that the observed temporal decrease in C_2+_ selectivity was primarily attributed to flooding rather than Cu relocation. Nevertheless, during the period before significant flooding began, the dynamically shifting electrolyte penetration front functioned as an effective TPB, maintaining high C_2+_ selectivity in the CO_2_RR. These results highlight the importance of considering not only the optimization of the initial catalyst morphology but also the structural changes and flooding behavior of the catalyst during CO_2_RR to improve catalyst selectivity and long-term stability at practical current densities.

## 4 Experimental Methods

### 4.1 Materials

All electrolytes were made by dissolving appropriate amounts of chemicals in Milli-Q water (Millipore, resistivity >18.2 MΩ cm). All chemicals were used without any further purification: KCl (99.5+ %), KHCO_3_ (99.5%), and KOH (85.0%) were purchased from FUJIFILM Wako Pure Chemical Corporation. AvCarb GDS2130 was used for a commercial carbon-based gas diffusion layer (GDL) with a microporous layer (MPL). A cation exchange membrane (CEM, Nafion 117) and an anion exchange membrane (AEM, Sustanion X37–50) were purchased from Fuel Cell Earth.

### 4.2 Preparation of Cu-GDEs

Cu­(*x*)-GDEs (thickness of Cu: *x* = 70, 300 nm), were prepared by RF magnetron sputtering of Cu onto a carbon-based MPL/GDL. In the case of flow cells, cathodes were prepared by affixing a PTFE seat with a 0.5 cm^2^ aperture onto the MPL/GDL through hot pressing (250 °C, 0.5 ton for 1 min) prior to Cu sputtering. This ensured that the electrode area was fixed.^15^ In the case of cross-sectional spectro-electrochemical cells for *in situ* SXFM, the cathodes were prepared by sputtering Cu over the entire surface of the MPL/GDL and then cutting them into pieces of 0.5 cm × 1.0 cm (0.5 cm^2^).

### 4.3 *Ex Situ* SEM-EDX

SEM images were obtained using a Hitachi S-4800 microscope. EDX elemental mappings were obtained utilizing an Oxford Instruments Aztec Ultim Max spectrometer.

### 4.4 Electrochemical Measurements for CO_2_RR

Electrochemical CO_2_RR experiments were performed in a flow cell configuration comprised of three distinct chambers: an anolyte chamber, a catholyte chamber, and a gas flow chamber (Figure S3). The cathode GDE was fixed between the catholyte chamber and the gas flow chamber, with the reverse side oriented toward the gas flow chamber and the catalyst side oriented toward the catholyte chamber. A Pt mesh was used as the counter electrode. The catholyte and anolyte chambers were separated by a CEM or AEM. The Nafion membrane (CEM) was completely hydrated by boiling it in deionized water, 5% hydrogen peroxide (H_2_O_2_) and 1 M sulfuric acid (H_2_SO_4_) at 80 °C for an hour each before use. The Sustanion membrane (AEM) was activated by immersion in 1 M KOH for 24 h before use. The catholyte chamber was equipped with an Ag/AgCl electrode (3 M KCl), which served as the reference electrode. The catholyte and anolyte were supplied through separate silicon tubes, each connected to a peristaltic pump. In the case of CO_2_RR with neutral electrolyte, a CEM was used and the catholyte was a 1 M KCl solution and the anolyte was a saturated KHCO_3_ solution. In the case of CO_2_RR with alkaline electrolyte, an AEM was used with a 1 M KOH solution as both catholyte and anolyte. The catholyte was pumped at a rate of 10 mL/min using a peristaltic pump and passed once through the chamber without circulation, while the anolyte was circulated through the chamber. A mass flow controller (MFC) was connected to a CO_2_ or an Ar gas cylinder to regulate the flow rate of 30 sccm into the gas flow chamber. All the electrochemical tests were conducted using a Biologic VSP potentiostat/galvanostat, which was equipped with a ± 2 A internal booster. The potential obtained in the flow cell were referenced to a reversible hydrogen electrode (RHE), and 85% *iR* compensation was performed based on the following equation: *E* (V vs RHE) = *E* (V vs Ag/AgCl) + 0.222 + 0.059 × pH + 0.85 × *iR*. where *i* is applying current, *R* is the resistance measured by electrochemical impedance spectroscopy (EIS) at OCP, and pH is the measured value of the bulk electrolyte before use. The current densities were calculated based on the geometric surface area.

The actual outlet gas flow rate of the flow cell was measured using a precision membrane flowmeter (SF-2U, Horiba). The gaseous products (H_2_, CH_4_, CO, C_2_H_4_) were quantified utilizing gas chromatography (dual column 990 Micro GC system, Agilent) with a thermal conductivity detector (TCD). The detection of H_2_, CH_4_, and CO was conducted using a Molsieve 5A (MS5A) column with an Ar as carrier gas. A PorraPlot Q (PPQ) column with a He carrier gas was employed for the detection of C_2_H_4_. The liquid products were evaluated using gas chromatography (GC2030, Shimadzu) with a flame ionization detector (FID) for alcohols (C_2_H_5_OH and C_3_H_7_OH) and high-performance liquid chromatography (HPLC, Nexcera Organic Acid System, Shimadzu) with a conductivity detector (CD) for organic acids (HCOOH and CH_3_COOH). The details of Faradaic efficiency calculations are given in Supporting Note S1 in the Supporting Information.

### 4.5 *Ex Situ* SXFM

SXFM experiments were conducted at the BL16XU of the SPring-8, Japan.^58^ The monochromatized X-ray beam, generated by an undulate and Si(111) double-crystal monochromator (DCM), was focused to a pinhole (virtual X-ray source) by a bend-cylindrical total reflection mirror. A focused micro-X-ray beam was formed by two elliptical total-reflection mirrors of Kirkpatrick-Baez configuration (KB mirrors) with a beam size of 1 μm square or less at the focal position (Figure S5b). The sample was mounted on the XYZ piezoelectric tables (PZT), which have a maximum travel of 250 μm and contraction at 10 V application. Subsequently, the X-ray microbeam was irradiated at the sample, and the X-ray fluorescence generated from the sample was detected by the Silicon Drift Detector (FAST SDD, Amptek), which was positioned diagonally upstream of the sample (Figure S5a). To prevent deformation of the cross-sectional Cu(300)-GDE samples during SXFM measurements, they were placed in a rigid plastic case. The *ex situ* SXFM images of the Cu(300)-GDE cross-section were obtained with an incident X-ray energy of 10.0 keV (over the Cu–K edge), with a scan area of 250 × 250 μm, with a pitch of 1 μm and 251 × 251 points, the scan rate of 10 Hz (resulting one image acquired in 1.74 h). The *ex-situ* SXFM images of the X-ray fluorescence intensity of Cu–Kα (Cu map) and K–Kα (K map corresponding to the KCl electrolyte) were subjected to analysis. The SXFM images were subsequently normalized by the incident X-ray intensity (*I*_0_). Due to the limitations of the scan area, it was necessary to obtain multiple SXFM images in the longitudinal direction, with some overlap in the scan area. Subsequently, the multiple images were superimposed and analyzed as a single longitudinal SXFM image.

### 4.6 *In Situ* XAS

XAS measurements were conducted at BL16B2 at SPring-8, Japan. Cu–K edge XAS was measured using a Si(111) monochromator. The beam size was 2.0 mm (H) × 1.0 mm (V). All data were recorded in fluorescence using the quick-XAS (QXAS) mode with a 25-element Ge solid-state detector (Canberra, 25SSD). A schematic and photograph of the *in situ* XAS measurement was shown in Figure S13. The configuration of the cell and the conditions for CO_2_RR were identical to those of the flow cell (Figure S3), but the outer wall of the gas chamber was replaced with a Kapton window to permit X-ray transmission. The X-rays were incident from the rear of the Cu(70)-GDE at a horizontal tilt of 45̊, and the Cu–K fluorescence X-rays were detected at the rear of the GDL. The *in situ* fluorescence XAS measurements were acquired on a repetitive and continuous basis, with each spectrum recorded over a period of 300 s. The Cu–K edge XANES data were processed using the LabView custom software and the Athena program of the Demeter data analysis package.^59^ To elucidate the role of Cu^1+^ during electrolysis, multipeak fitting of the first derivative XANES spectra was conducted based on the energy positions of Cu^0^ (8978 eV) and Cu^1+^ (8980 eV).

## Supplementary Material



## Notes

Quantification of CO_2_RR products, *ex situ* X-ray CT procedure and images, schematic diagram of the flow cell for CO_2_RR, *in situ* SXFM experimental setup and results, *in situ* XAS experimental setup, multipeak-fitting of the first derivative XANES spectra, *ex situ* STEM-EDX images, ICP-MS results of the electrolytes, time-dependence of FEs during CO_2_RR, ECSA measurements (PDF)
