# Supplementary material for: Dynamic Relocation of Copper Catalysts in Gas Diffusion Electrodes during CO2 Electroreduction
Source: J Am Chem Soc. 2025 Jun 25;147(27):24103–12. doi: 10.1021/jacs.5c07944 (PMC12257527; doi:10.1021/jacs.5c07944)
Supplement: Supplementary file 1 [file ja5c07944_si_001.pdf]

Supporting Information

Dynamic Relocation of Copper Catalysts in Gas Diffusion Electrodes during CO_2 Electroreduction

Daiko Takamatsu*[a], Naoto Fukatani[a], Akio Yoneyama[a], Tatsumi Hirano[a], Kakuro Hirai[a], Shin Yabuuchi[a], Koichi Watanabe[a], Kazuhide Kamiya[b][c], and Shuji Nakanishi[b][c]

[a] Center for Exploratory Research, Research & Development Group, Hitachi, Ltd., 2520, Akanuma, Hatoyama-machi, Saitama 350-0395 (Japan)

[b] Research Center for Solar Energy Chemistry, Graduate School of Engineering Science, Osaka University, 1-3, Machikaneyama, Toyonaka, Osaka 560-8531 (Japan)

[c] Innovative Catalysis Science Division, Institute for Open and Transdisciplinary Research Initiatives (ICS-OTRI), 2-1, Yamada-oka, Suita, Osaka 565-0871 (Japan)

*Corresponding author: daiko.takamatsu.hu@hitachi.com (D.T.)

Contents

Supporting Notes S1-S3

Figures S1-S22

Table S1

Supporting Note.

S1. Faradaic efficiency calculations.

Gaseous products:

The gaseous products were quantified utilizing gas chromatography (GC) with a thermal conductivity detector (TCD). The quantity of each gaseous product (mol) can be determined from the peak areas of the GC and the calibration curves of the TCD. The number of electrons n_{e-} required to produce a molecule of H_2, CO, CH_4, and C_2H_4 are 2, 2, 8, and 12, respectively. The total number of moles of electrons required to produce the reduction product y (mol) is given by the following equation:

$$e_{output} = y \times n_{e-} \qquad (1)$$

The measured charge can be determined by applying current i (A) and the time required to fill the sampling loop t (sec). This can be expressed as $Q = i \times t$. The total number of moles of electrons measured during the sampling period is given by the following equation:

$$e_{input} = Q/F = (i \times t)/F \qquad (2)$$

where F is Faraday constant: 96485 (C/mol) and Q is the measured charge (C). The Faradaic efficiency (FE) of the gaseous product can be calculated as follows:

$$FE_{gas\ product} = (e_{output} / e_{input}) \times 100\% \qquad (3)$$

Liquid products:

The liquid products were evaluated using GC with a flame ionization detector (FID) for alcohols and high-performance liquid chromatography (HPLC) with a conductivity detector (CD) for organic acids. From the data obtained from the peak areas and calibration curves, the concentration of each component in the sampling liquid volume can be determined. Subsequently, the content y (mol) of each component in the total catholyte recovery liquid V (mL) collected from the electrolysis cell after constant-current CO_2RR is determined. The number of electrons n_{e-} required to produce a molecule of HCOOH, CH_3COOH, C_2H_5OH, and C_3H_7OH are 2, 8, 12, and 18, respectively. The total number of moles of electrons required to produce y (mol) of the reduction product is given by the following equation:

$$e_{output} = y \times n_{e-} \qquad (4)$$

The total number of moles of electrons supplied when applying current i (A) for time t (sec) is given by the following equation:

$e_{input} = Q/F = (i \times t)/F$ (5)

The FE of the liquid product is calculated using the following equation:

$FE_{liquid\ product} = (e_{output} / e_{input}) \times 100\ \%$ (6)

S2. *Ex situ* X-ray computed tomography (CT)

Ex situ X-ray CT experiments were performed at BL14B of the Photon Factory (PF), Japan and at BL07 of the SAGA-LS, Japan, respectively. For the CT at the PF BL14B, the energy of the monochromatic synchrotron radiation (SR) used was set to 15 keV, the exposure time for obtaining a projection images was 2 s, and the number of projections was set to 2000 images/360°. The projection images were captured by an X-ray microscopic camera (kenvy2) made in house[S1], with a x5 objective lens, a pixel binning of 1, an effective pixel size of 1.3 μm, 2048 x 2048 pixels and a field of view of 2.6 mm square. The phosphor used was $Gd_3Al_2Ga_3O_{12}$:Ce (GAGG).

The spectral CT of the Cu element was carried out at the SAGA-LS BL07 using the dual-energy CT method, which can visualize the spatial distribution of the Cu by calculating the difference between two CT images acquired at energies around the absorption edge of Cu-K. The energies for the spectral CT of the Cu were set at 8.82 keV and 9.11 keV. The exposure time to obtain a projection image was 2 s, and the number of projections was set at 2000 images/360°. The projection images were detected by kenvy2, with a x5 objective lens, a pixel binning of 2, an effective pixel size of 2.6 μm, 2048 x 2048 pixels and a field of view of 2.6 mm square. The phosphor used was CsI.

Each CT cross-sectional image was reconstructed using SAKAS (SAGA Light Source data Karte System[S2]) software with a filtered-back projection method using a Shepp-Logan filter.

S3. *In situ* scanning X-ray fluorescence microscopy (SXFM)

In situ SXFM measurements were carried out at the BL16XU of the SPring-8, Japan and at the BL07 of the SAGA-LS, Japan. *In situ* SXFM was conducted using a spectro-electrochemical cell, which enabled the observation of the Cu catalyzed gas diffusion electrode (Cu-GDE) cross-section during electrolysis (Figure S8). The cell was comprised of a 0.5 x 1.0 cm^2 area of the Cu(300)-GDE cathode, a Pt-plated Ti porous transport layer (PTL) anode, and a cation exchange membrane (CEM) or anion exchange membrane (AEM), which enabled the introduction of gas and electrolyte through Au current collectors with flow channels. A supply of CO_2 or Ar gas was introduced into the cathode channel at a rate of 30 sccm, while an electrolyte (0.1M $KHCO_3$ for neutral condition and 1M KOH for alkaline condition) was introduced into the anode channel at a flow rate of 10 ml/min. At the BL16XU of the Spring-8 (Figure S8a), the *in situ* SXFM images of Cu-fluorescence intensity (Cu-map) were acquired with an incident X-ray energy of 10.0 keV, a scan range of 250 x 250 μm, a 5 μm pitch, 51 x 51 points, and a scan rate of 10 Hz (resulting in one image acquired every 261 seconds). At the BL07 of the SAGA-LS (Figure S8b), the *in situ* SXFM images of Cu-map were acquired with an incident X-ray energy of 10.0 keV (pseudo-monochromatic beam), a scan range of 250 x 50 μm, a 2.5 μm pitch, 101 x 11 points, and a scan rate of 100 ms/pulse (resulting in one image acquired every 153 seconds).

In situ SXFM at Spring-8 BL16XU: in neutral electrolyte under CO_2 or Ar supply

In situ SXFM observation of the Cu(300)-GDE cross-sectional region during electrolysis in neutral electrolyte under CO_2 or Ar supply was performed at BL16XU of the Spring-8 (Figure S8a). Figure S9a shows representative Cu maps acquired during linear sweep voltammetry (LSV) from 0 to -4.8 V (scan rate: 2 mV/sec) under CO_2 supply. No notable alterations in the Cu catalyst layer (CL) configuration were discerned at relatively low current densities (#1-#7). At current densities exceeding ≥ -200 mA/cm^2 (from #8 onward), dispersed Cu was observed within the microporous layer (MPL). Figure S9b illustrates line profiles along the x-axis, averaged on the z-axis, of the Cu map obtained at open circuit potential (OCP) before LSV (#0) and after LSV (#12). At OCP following the applied current (#12), a distinct tail shape emerged within the MPL, which was not present prior to the LSV (#0). The alterations in the CL area and

CL width, as determined from the line profiles shown in Figure S9b, are presented in conjunction with the LSV curve in Figure S9c. Here, the term "CL area" is defined as the integrated intensity of the line profile representing the amount of Cu fluorescence X-ray detected, and the term "CL width" is defined as the maximum width at the baseline of the Cu profile representing the depth of Cu migration into the MPL. As shown in Figure S9c, the CL area tended to decrease with repeated small increases and decreases at relatively low current densities (#1-#7), indicating attenuation of Cu fluorescence X-rays due to the electrolyte penetration into the CL (Figure S10b). The CL area switched to an increase at current densities above 200 mA/cm^2 (#8-#9), suggesting that the migrated Cu within the MPL enhances the total Cu fluorescence X-rays (Figure S10c). The CL width remained constant at relatively low current densities (#1-#7), subsequently widening at current densities above $\geq$ 200 mA/cm^2 (#8-#9), and then remaining constant at OCP after LSV (#10-#12). This suggests that the depth of Cu migration into the MPL is dependent on the quantity of charge passed, which is consistent with the *ex situ* SXFM results (Figure 2c in the manuscript). The results of the *in situ* SXFM analysis conducted under the same LSV process with Ar supply are presented in Figure S9d. Under Ar supply, the CL area decreased significantly even at low current density (#6), remained reduced during the LSV (#6-#8), and increased slightly at OCP (#9-#10) (Figure S9d). This suggests that flooding occurs due to the electrowetting effect when a negative voltage is applied and that the Cu fluorescence X-rays are attenuated (Figure S10d). When the current application is stopped, the degree of flooding decreases, so the Cu fluorescence X-rays from CL increase. The CL width remained unchanged during the LSV under Ar supply, indicating that Cu migration to the MPL did not occur (Figure S9d).

After LSV, *in situ* SXFM was performed during chronopotentiometry (CP)-OCP cycles, where CP and OCP were repeated with stepwise increasing current, with CO_2 supply (Figure S11) and Ar supply (Figure S12), respectively. During CP-OCP cycles, the changes in the CL area and CL width were more pronounced than during LSV. This is because the current densities during CP are sufficiently high ($\geq$ 200 mA/cm^2) and the time for each current application is longer than during LSV. As a result, there were significant differences in the Cu maps after the CP-OCP cycles between CO_2 or Ar supply. Figure S9e

shows the line profiles along the x-axis, averaged on the z-axis, of the Cu map obtained at OCP before and after a series of electrolysis (LSV and CP-OCP cycles) with CO_2 and Ar supply. After electrolysis under CO_2 supply, Cu migrated into the MPL and its shape changed significantly (Figure S9e upper). In contrast, after electrolysis under Ar supply, Cu remained on the MPL surface, and its shape changed only slightly (Figure S9e lower). In both cases of CO_2 supply and Ar supply, the CL area after a series of electrolysis was smaller than the initial state, suggesting the dissolution of Cu.

***In situ* SXFM at SAGA-LS BL07: in neutral or alkaline electrolyte under CO_2 supply**

In situ SXFM observation of the Cu(300)-GDE cross-sectional region during electrolysis in neutral or alkaline electrolyte under CO_2 supply was performed at BL07 of the SAGA-LS (Figure S8b). Figure S20a shows time-evolution of Cu intensity profiles along with x axis (integrated in z axis) acquired during CP-OCP cycles with current density incremented in steps of -100, -200, -300, -400, -600, and -800 mA/cm^2 under CO_2 supply. Comparing the degree of Cu migration depending on the initial pH of the electrolyte, the depth of Cu migration to MPL was more pronounced in the alkaline electrolyte (Figure S21b) than in the neutral electrolyte (Figure S21a). As a result, the Cu map after the CP-OCP cycles showed that Cu was more widely distributed in the alkaline electrolyte (Figure S22b) than in the neutral electrolyte (Figure S22a).

References

(S1) Yoneyama, A.; Baba, R.; Kawamoto, M. Quantitative analysis of the physical properties of CsI, GAGG, LuAG, CWO, YAG, BGO, and GOS scintillators using 10-, 20- and 34-keV monochromated synchrotron radiation. *Optical Materials Express*, **2021**, *11*, 398.

(S2) https://www.saga-ls.jp/main.php/3092.html# gsc.tab=0

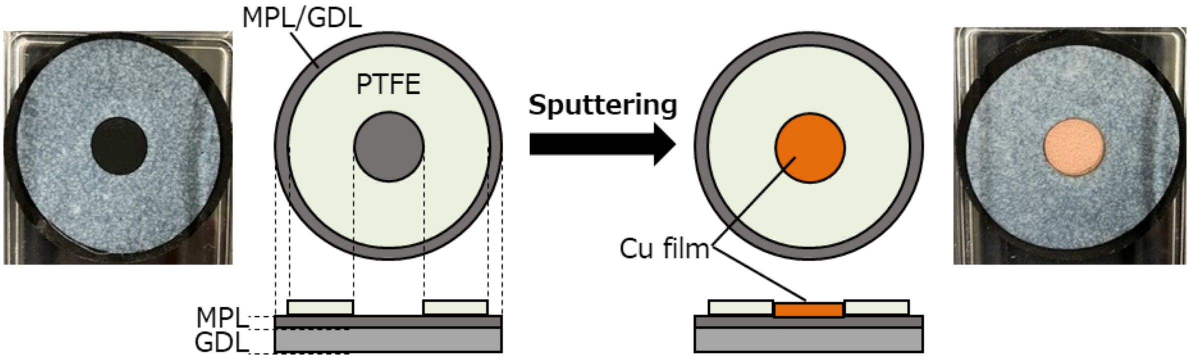

Figure S1 Schematic and photographs of the preparation of Cu(*x*)-GDEs (*x* = 70, 300 nm) used as cathodes for flow cells. Cu(*x*)-GDEs were prepared by magnetron sputtering of Cu on a commercial carbon-based gas diffusion layer (GDL) with a microporous layer (MPL). The catalyst area was defined by thermo-compressed PTFE (the geometric area of the hole is 0.5 cm^2).

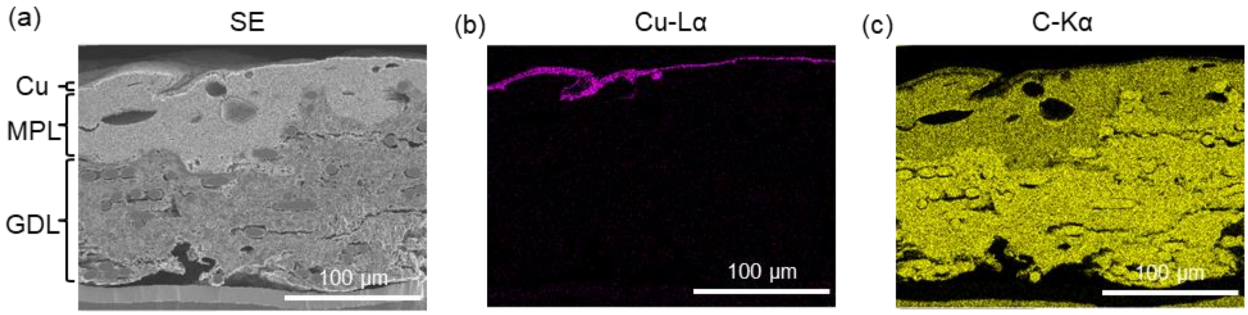

Figure S2 (a) Cross-sectional SEM images (x 500) of the prepared Cu(300)-GDE (pristine) and EDX mapping of (b) Cu and (c) C elements corresponding to (a). SE: secondary electron image, EDX: energy dispersive X-ray spectroscopy.

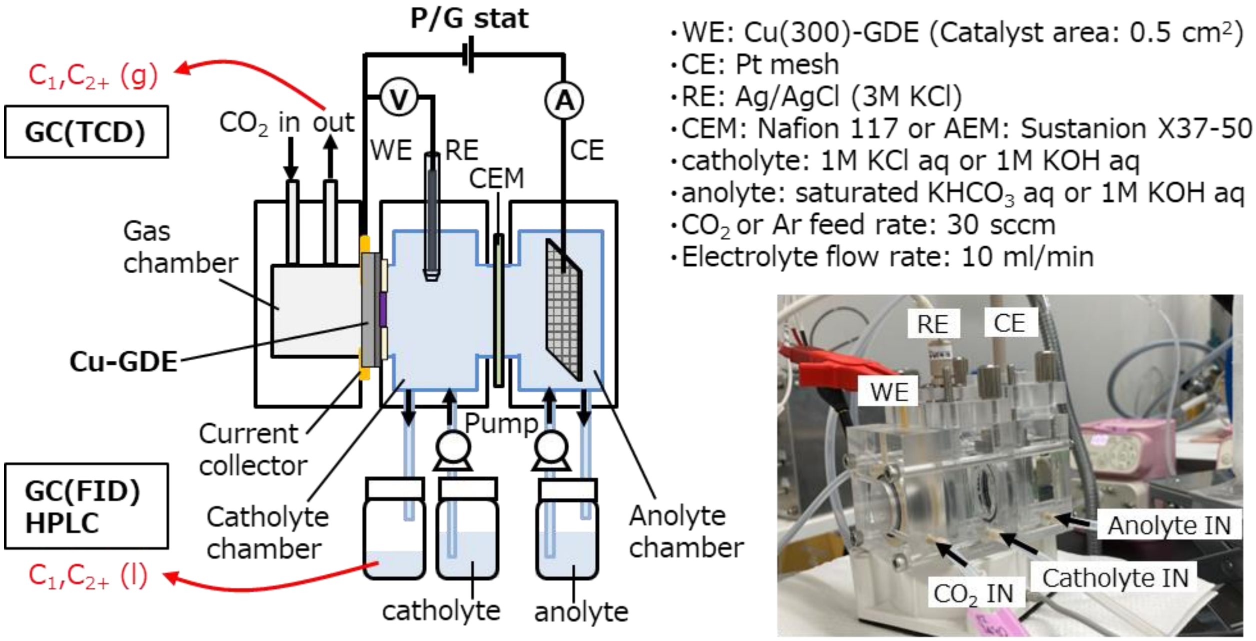

Figure S3 Schematic and photograph of a flow cell for CO_2RR. P/G stat: Potentiostat/Galvanostat.

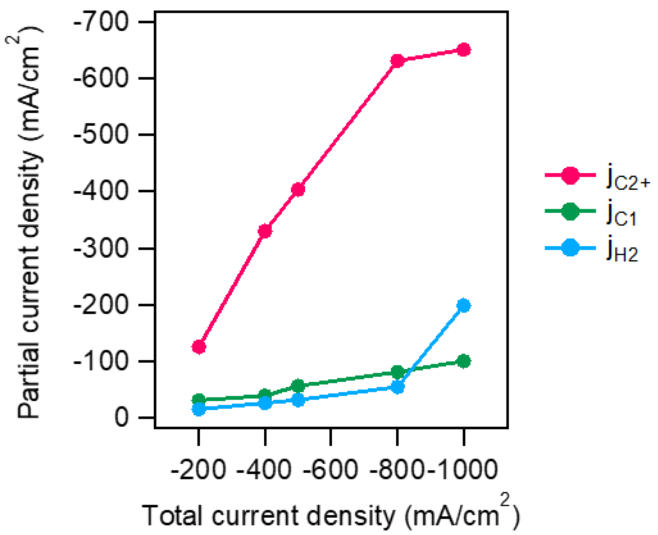

Figure S4 (a) The partial current density for C_{2+}, C_1, and H_2 on Cu(300)-GDE in 1M KCl calculated from Figure 1a of the manuscript.

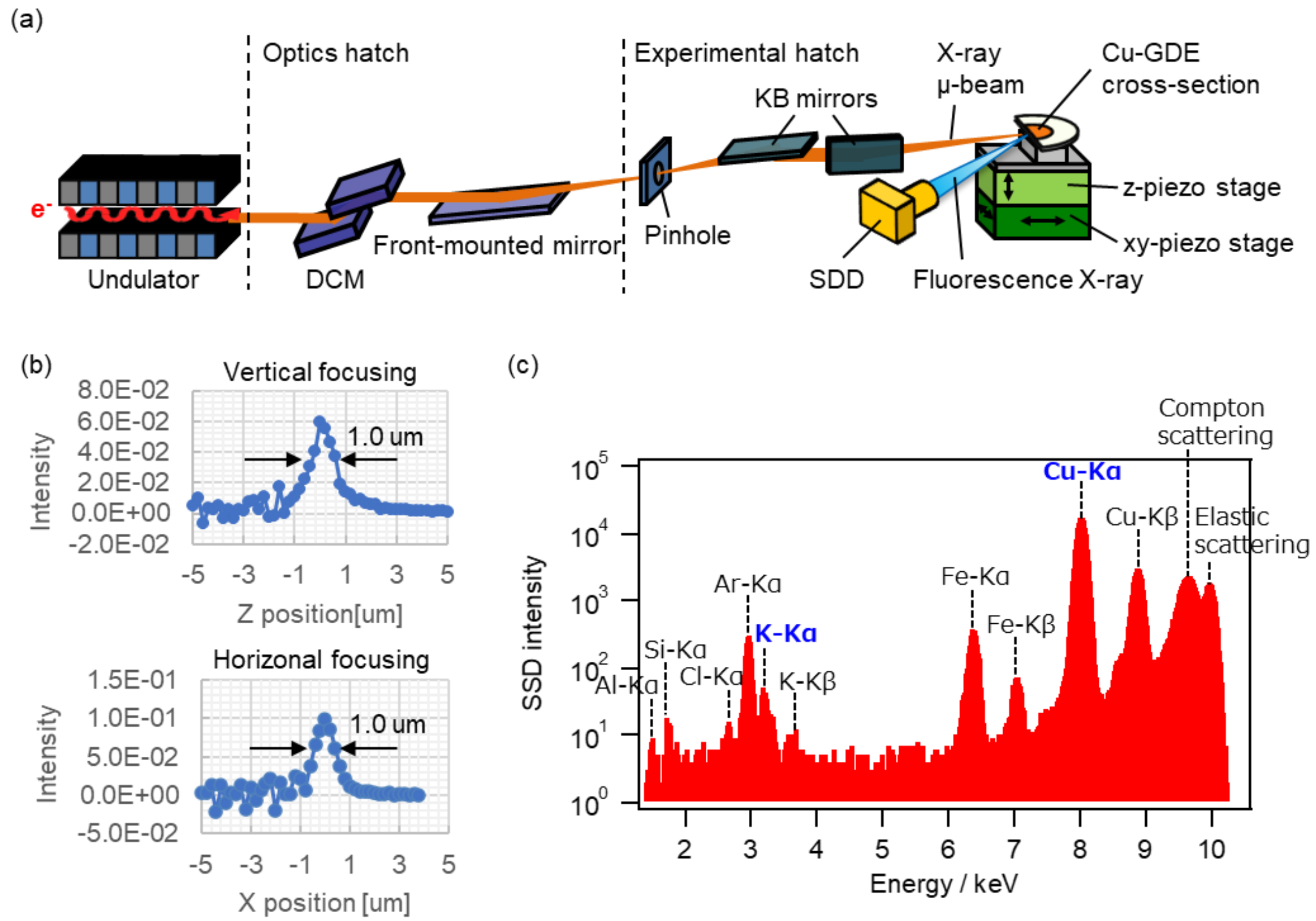

Figure S5 (a) Schematic of the SXFM measurement system at SPring-8 BL16XU for *ex situ* observation of the GDE cross-sectional sample. (b) Two-dimensional intensity profile of the incident X-ray obtained at the focal position. (c) X-ray fluorescence intensity spectrum of SDD acquired at the Cu-catalyst position in the sample.

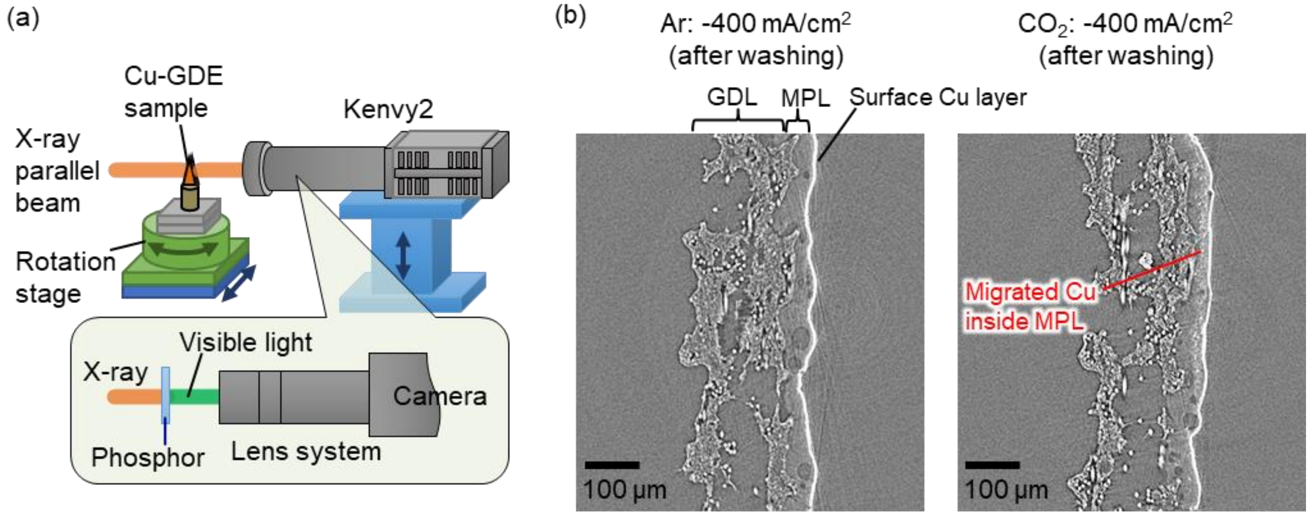

(c)

Methods	Detection depth
SEM-EDX (Accelerating voltage: 5 kV)	~1 µm
SXFM (Incident X-ray energy: 10 keV)	5 µm (for Cu)<br>2000 µm (for C)

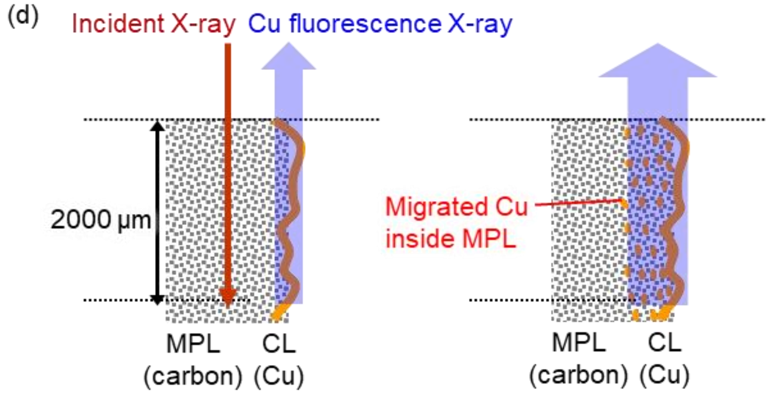

Figure S6 (a) Schematic of *ex situ* X-ray CT measurement system at PF BL14B. (b) Cu(300)-GDE cross-sectional samples (after washing): after CP at -400 mA/cm^2 for 20 min under Ar or CO_2 supply, respectively. Washing procedure: GDE was washed with a solvent to remove the precipitated salts and water inside the electrode and dried. (c) Approximate detection depth for SEM-EDX and SXFM. For SXFM, the X-ray attenuation length (E = 10 keV) of MPL/GDL (Carbon) and CL (Cu) are shown. (d) Schematic illustration of the reason why the width of the Cu layer appears wider in SXFM than the actual film thickness.

The X-ray attenuation length of a solid was obtained from the following reference: B. L. Henke, E. M. Gullikson, J. C. Davis, *Atomic Data and Nuclear Data Tables*, **1993**, *54*, 181-342.

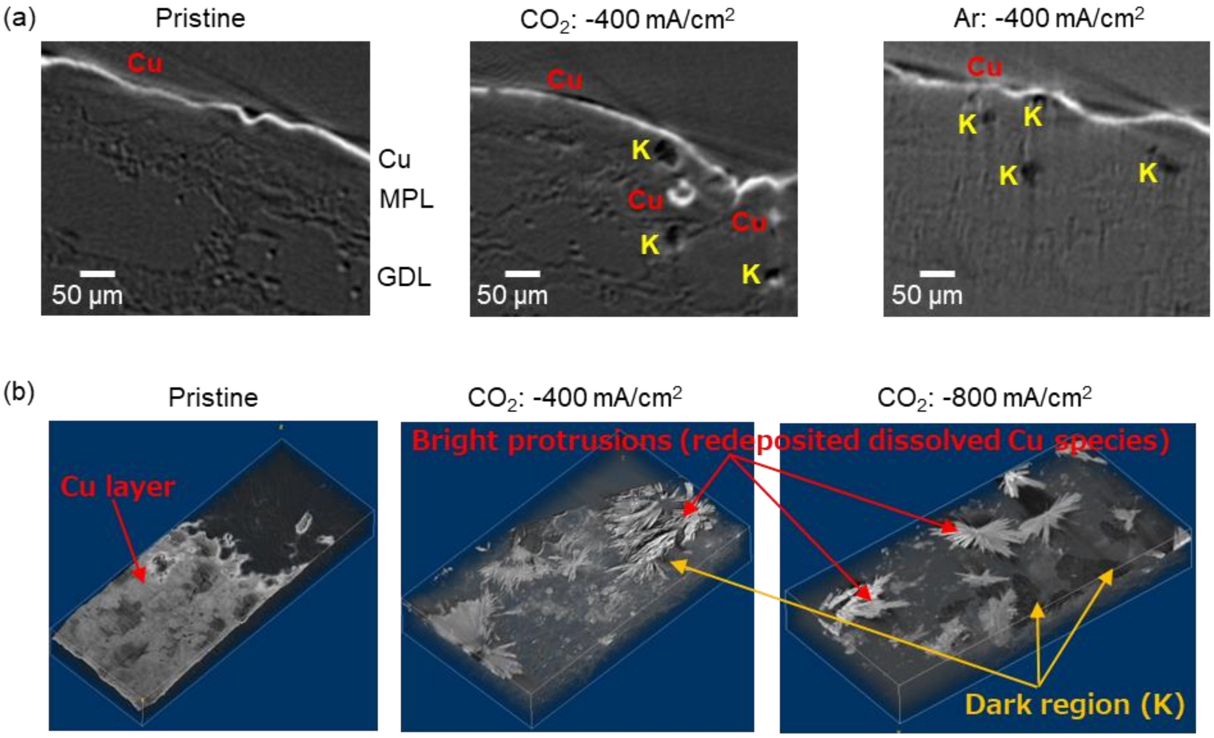

Figure S7 (a) *Ex situ* spectral X-ray CT images around Cu-K edge obtained at SAGA-LS BL07 of Cu(300)-GDE cross-sectional samples: as-prepared (pristine), after CP at -400 mA/cm^2 for 20 min under CO_2 supply (no washing), and after CP at -400 mA/cm^2 for 20 min under Ar supply (no washing). (b) Spectral CT images of the Cu(300)-GDE surfaces: as-prepared (pristine), after CP at -400 mA/cm^2 for 20 min under CO_2 supply (no washing), and after CP at -800 mA/cm^2 for 20 min under CO_2 supply (no washing).

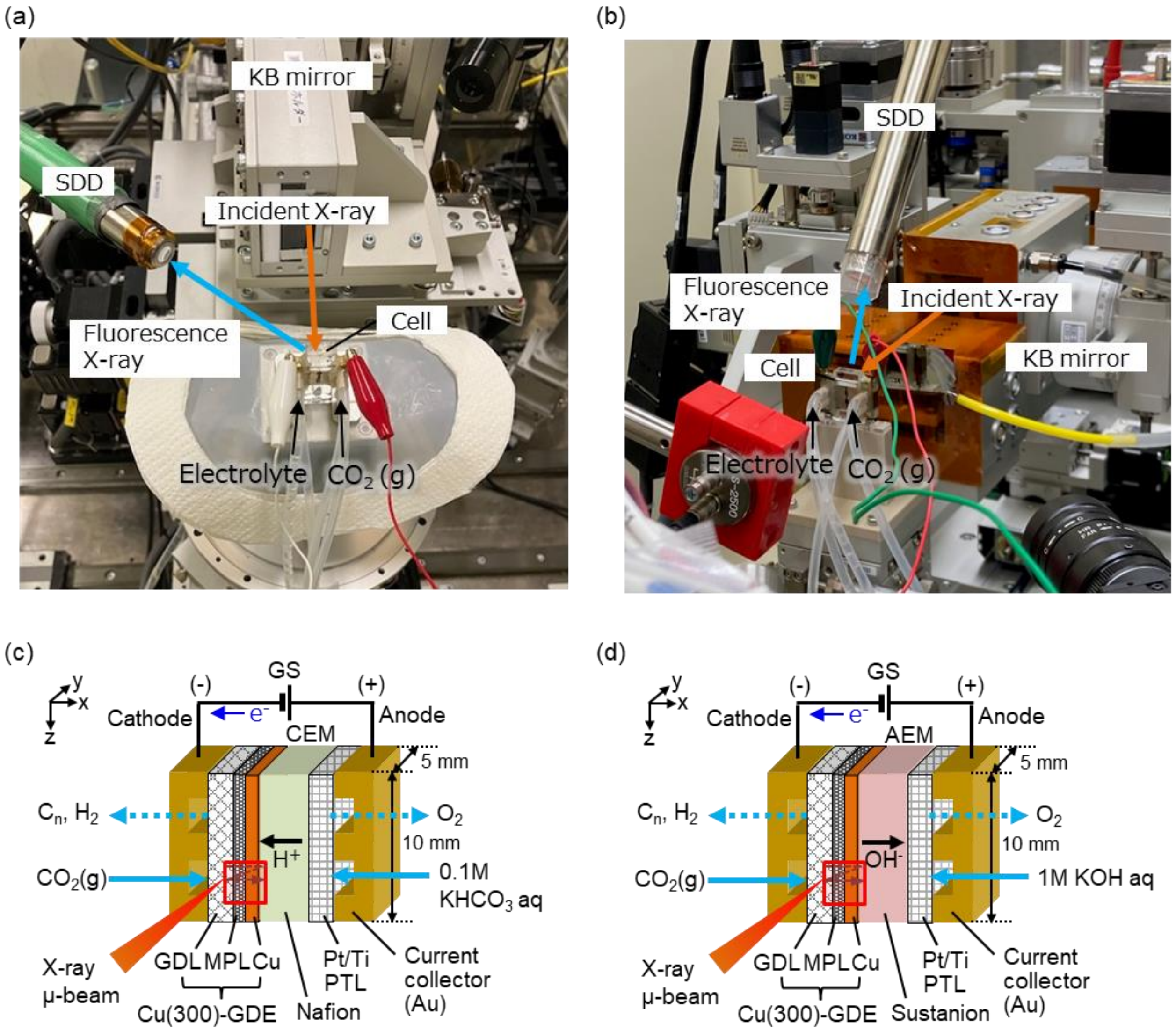

Figure S8 (a)(b) Photograph of *in situ* SXFM measurement: (a) at BL16XU of Spring-8 and (b) at BL07 of SAGA-LS. (c)(d) Schematic of a spectro-electrochemical cell consisting of a Cu(300)-GDE cathode, Pt/Ti PTL anode, and polymer electrolyte membrane: (c) CEM is used for neutral electrolyte, (d) AEM is used for alkaline electrolyte. Continuous SXFM images of the GDE cross-section in the red square region were analyzed. GS: Galvanostat.

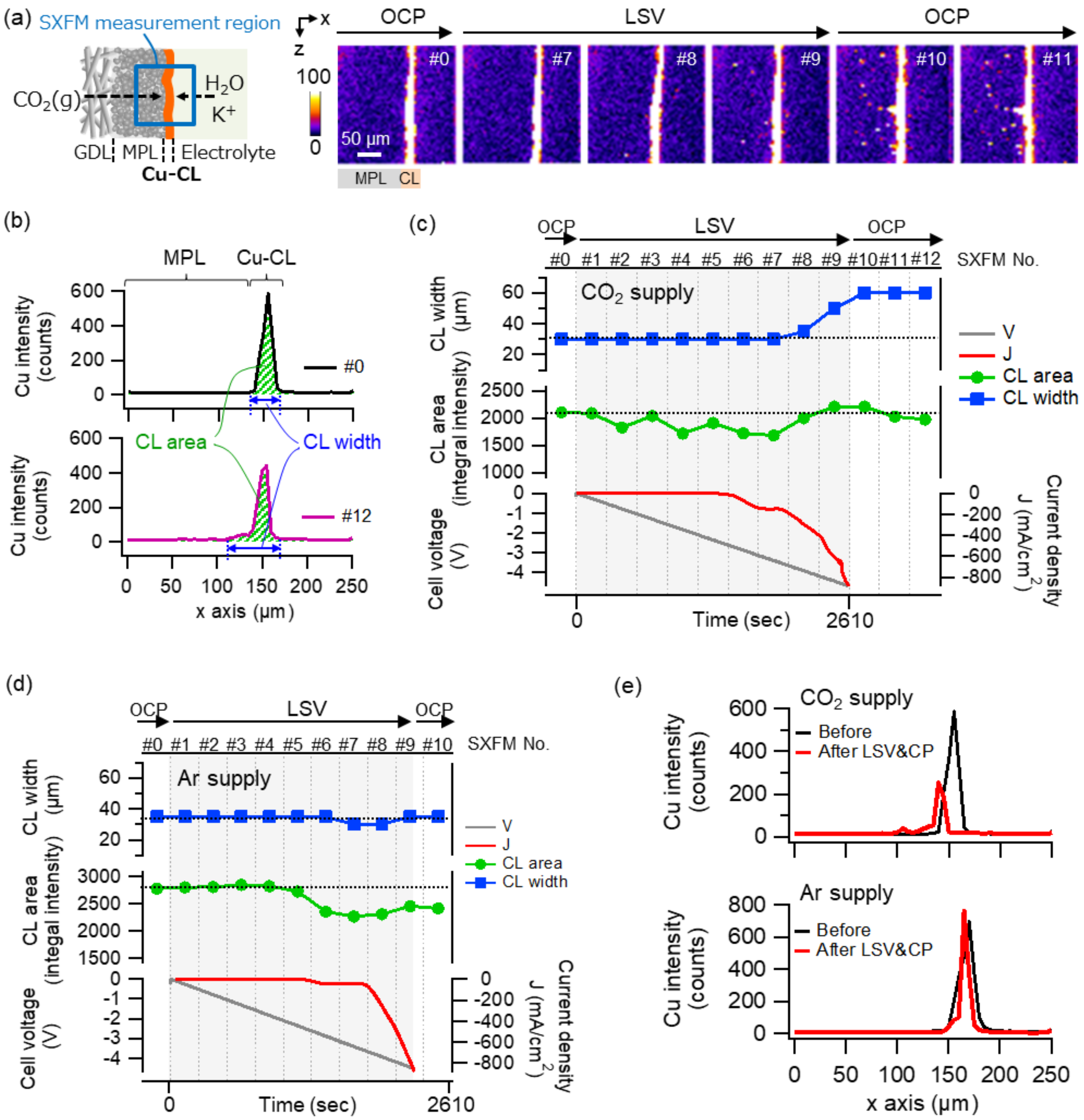

Figure S9 *In situ* SXFM results obtained at SPring-8 BL16XU. (a) Schematic cross-section of the cell for SXFM measurement region. Continuous SXFM images in the square region were analyzed. Extracted Cu maps of Cu(300)-GDE cell during LSV under CO_2 supply. (b) Extracted line profiles along x-axis averaged on z-axis and (c) corresponding LSV curve, CL area (integral intensity), and CL width during LSV under CO_2 supply. (d) The LSV curve, CL area, and CL width results during LSV under Ar supply. (e) The line profiles along x-axis averaged on z-axis obtained before and after electrolysis under CO_2 or Ar supply.

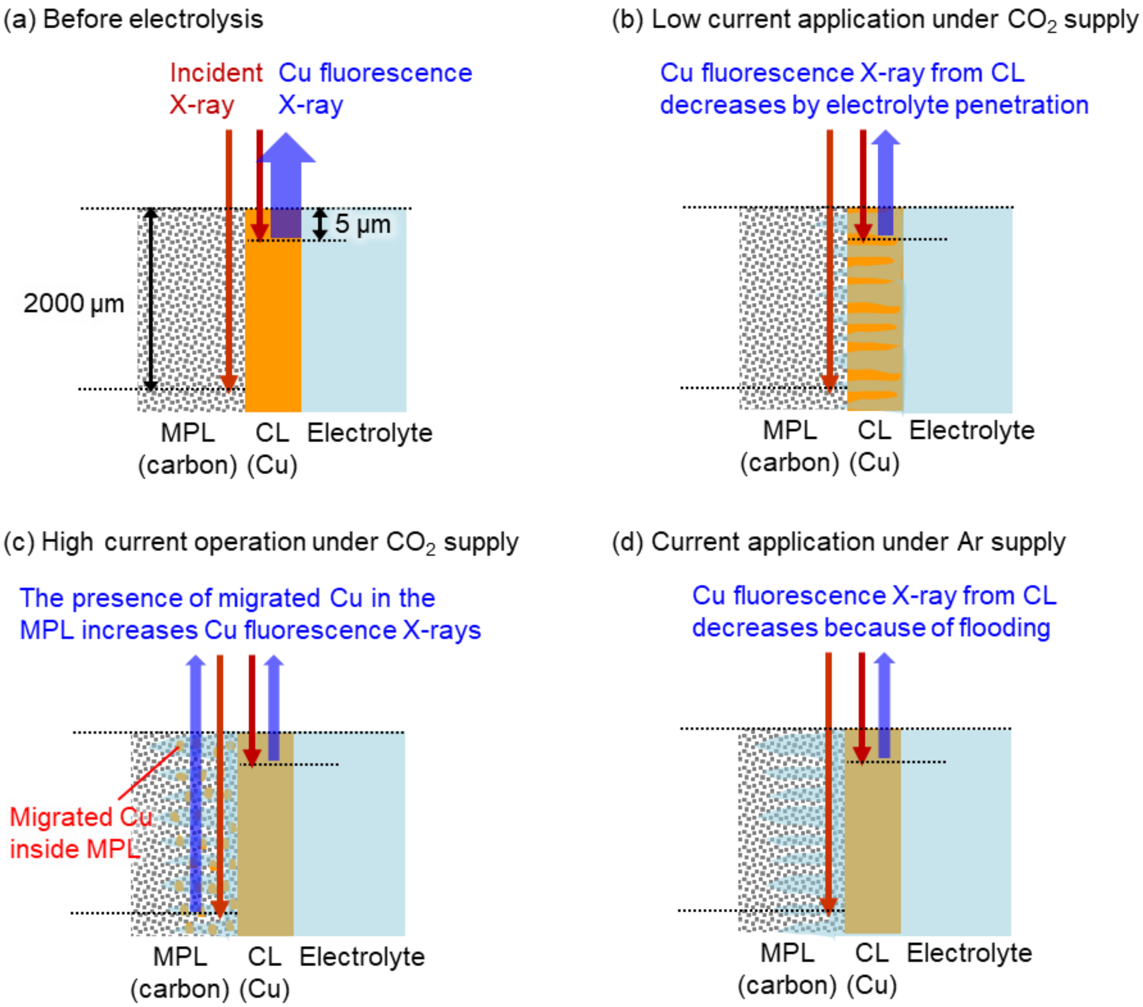

Figure S10 Schematic illustration of the difference in X-ray attenuation length (E = 10 keV) for MPL (porous carbon) and CL (Cu). (a) before electrolysis (electrolyte soaked), (b) low current application under CO_2 supply, (c) high current application under CO_2 supply, (d) current application under Ar supply.

The X-ray attenuation length of a solid was obtained from the following reference: B. L. Henke, E. M. Gullikson, J. C. Davis, *Atomic Data and Nuclear Data Tables*, **1993**, *54*, 181-342.

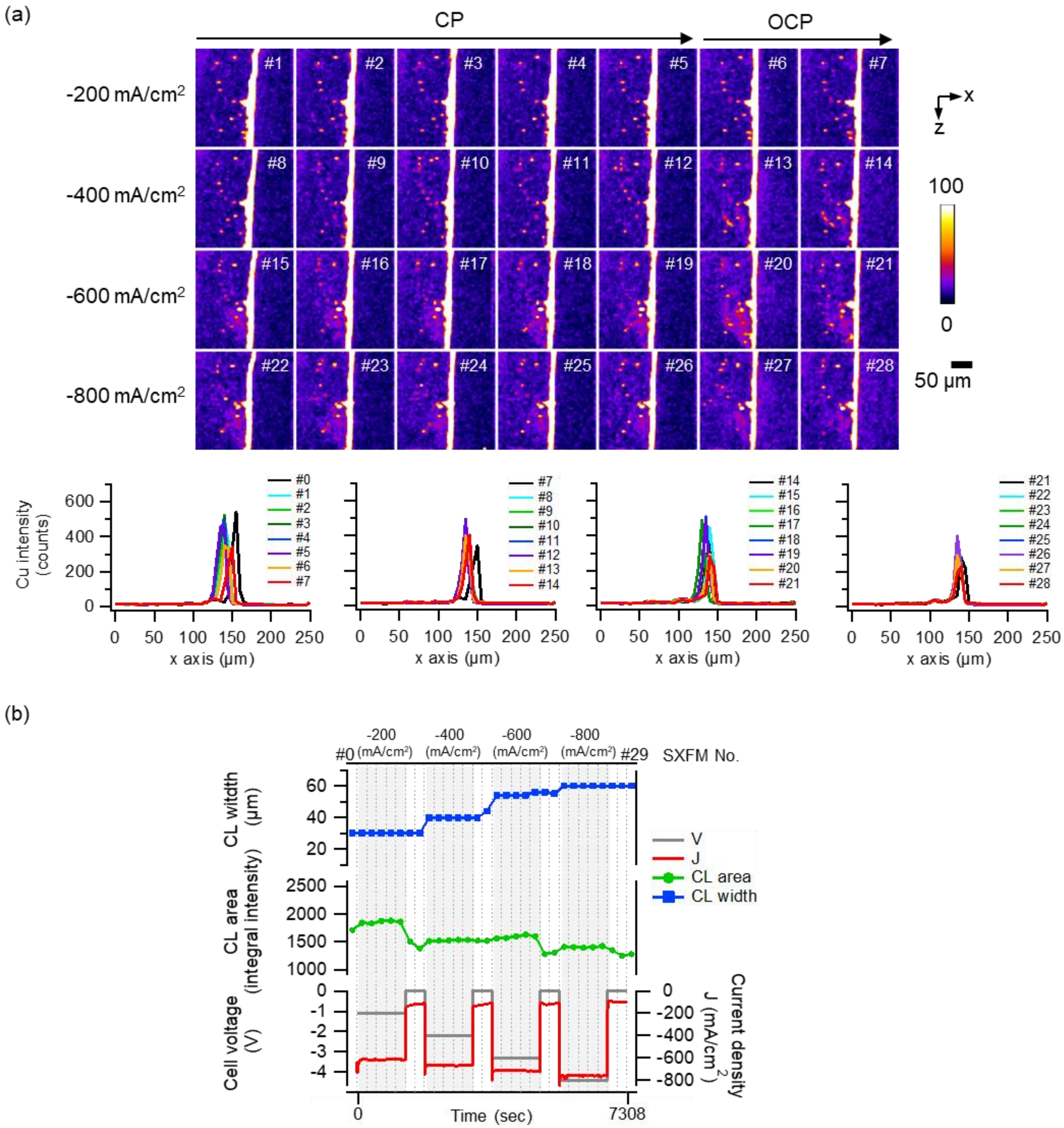

Figure S11 *In situ* SXFM images (Cu-maps) of Cu(300)-GDE cell under CO_2 supply obtained at SPring-8 BL16XU. (a) Cu-maps and their line profiles along x-axis averaged on z-axis obtained during CP-OCP cycles. (b) J-V curve, CL area, and CL width during CP-OCP cycles.

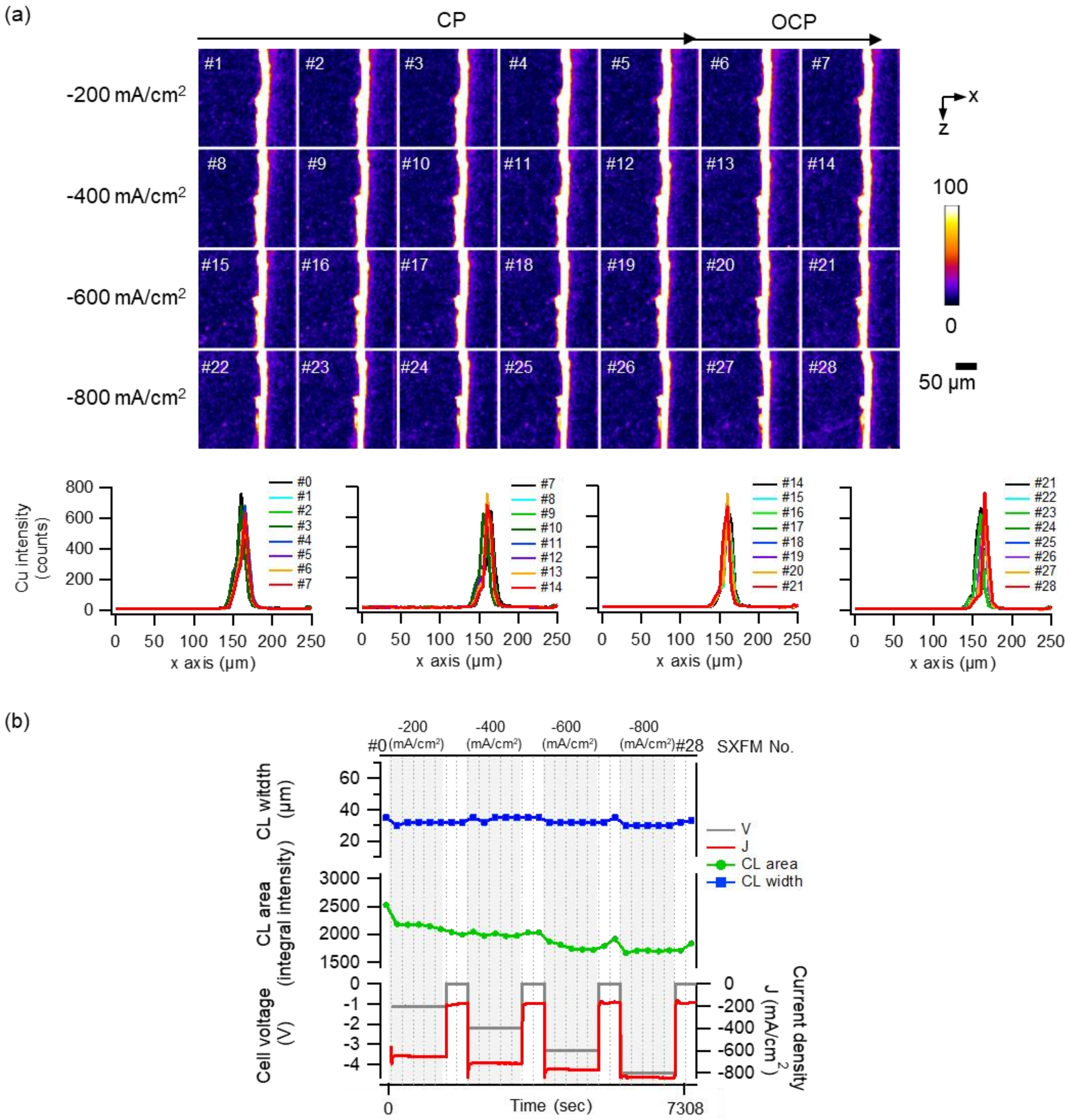

Figure S12 *In situ* SXFM images (Cu-maps) of Cu(300)-GDE cell under Ar supply obtained at SPring-8 BL16XU. (a) Cu-maps and their line profiles along x-axis averaged on z-axis obtained during CP-OCP cycles. (b) J-V curve, CL area, and CL width during CP-OCP cycles.

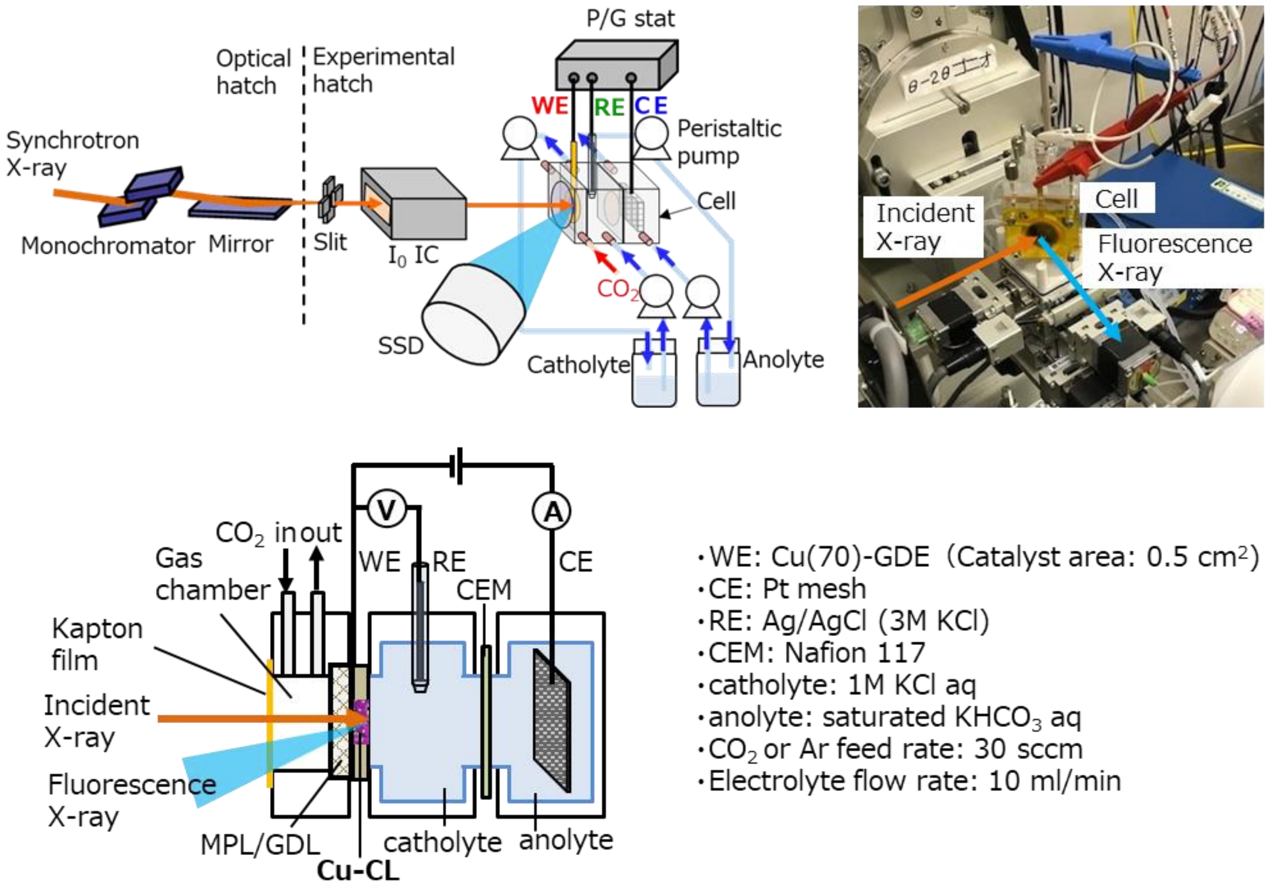

Figure S13 Schematic and photograph of the flow cell modified by Kapton window with Cu(70)-GDE cathode for *in situ* fluorescence XAS measurement at SPring-8 BL16B2.

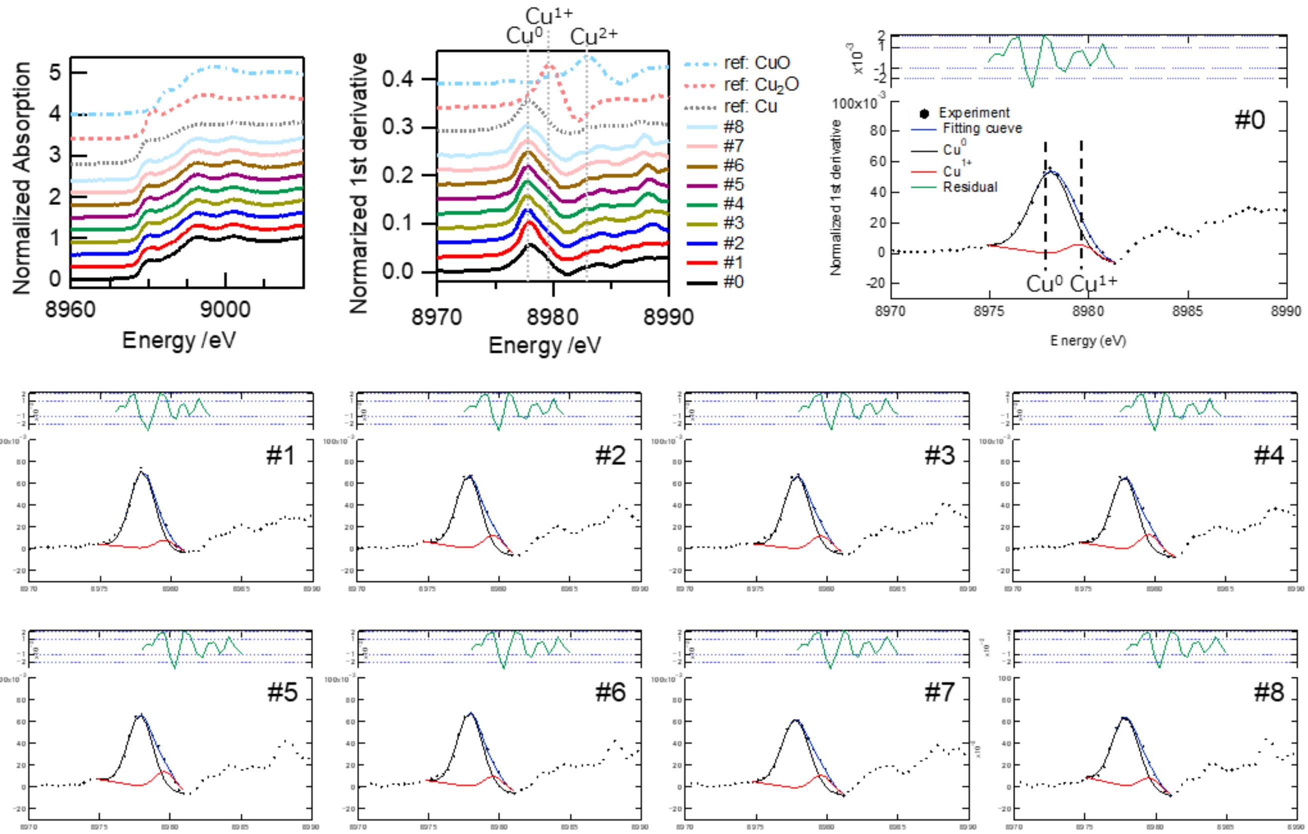

Figure S14 *In situ* normalized Cu-K edge XANES spectra and normalized 1st derivative spectra of the Cu(70)-GDE during CP -400 mA/cm^2 and OCP under CO_2 supply, together with the standard samples. Multi-peak-fitting results are shown in Fig. 3b in the manuscript.

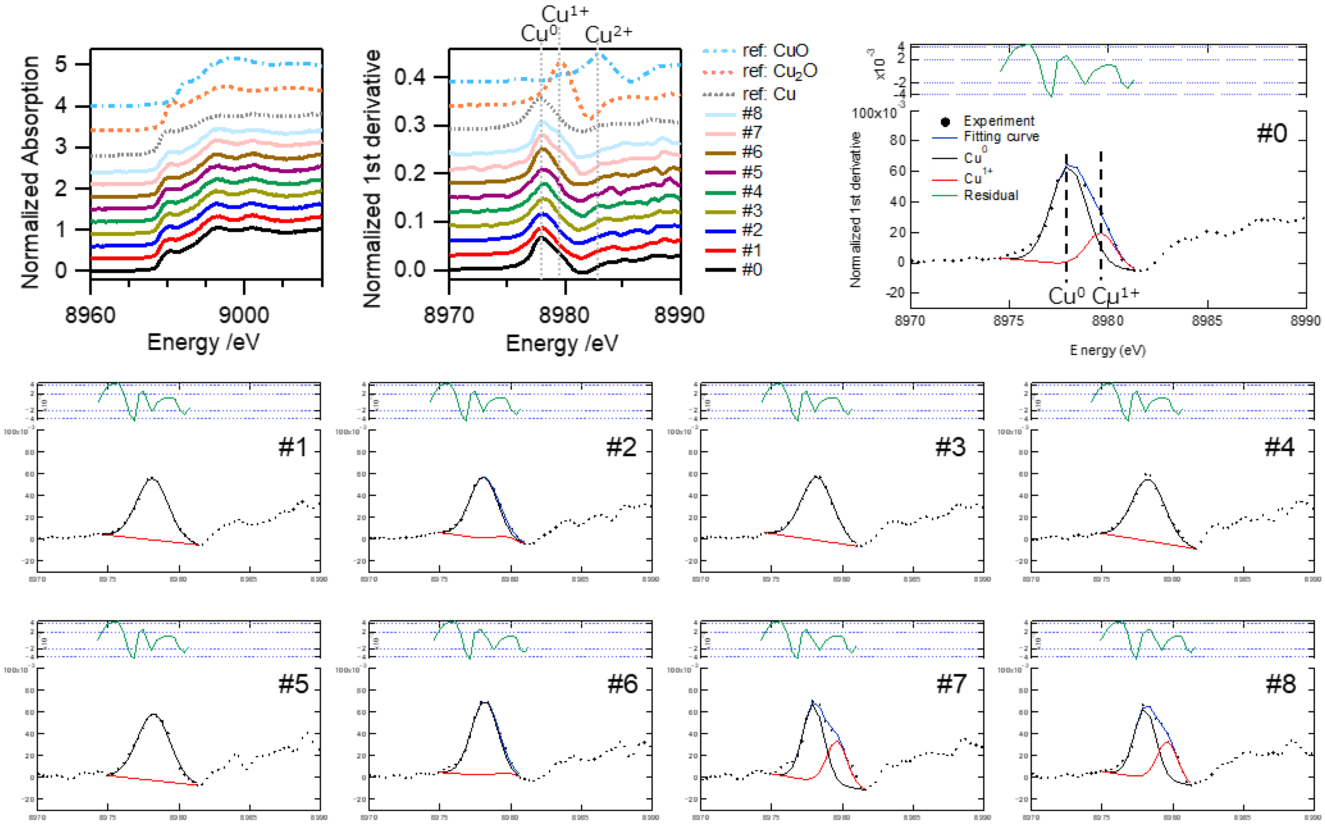

Figure S15 *In situ* normalized Cu-K edge XANES spectra and normalized first derivative XANES spectra of the Cu(70)-GDE cell during CP -400 mA/cm^2 and OCP under Ar supply, together with the standard samples. Multi-peak-fitting results are shown in Figure 3b in the manuscript.

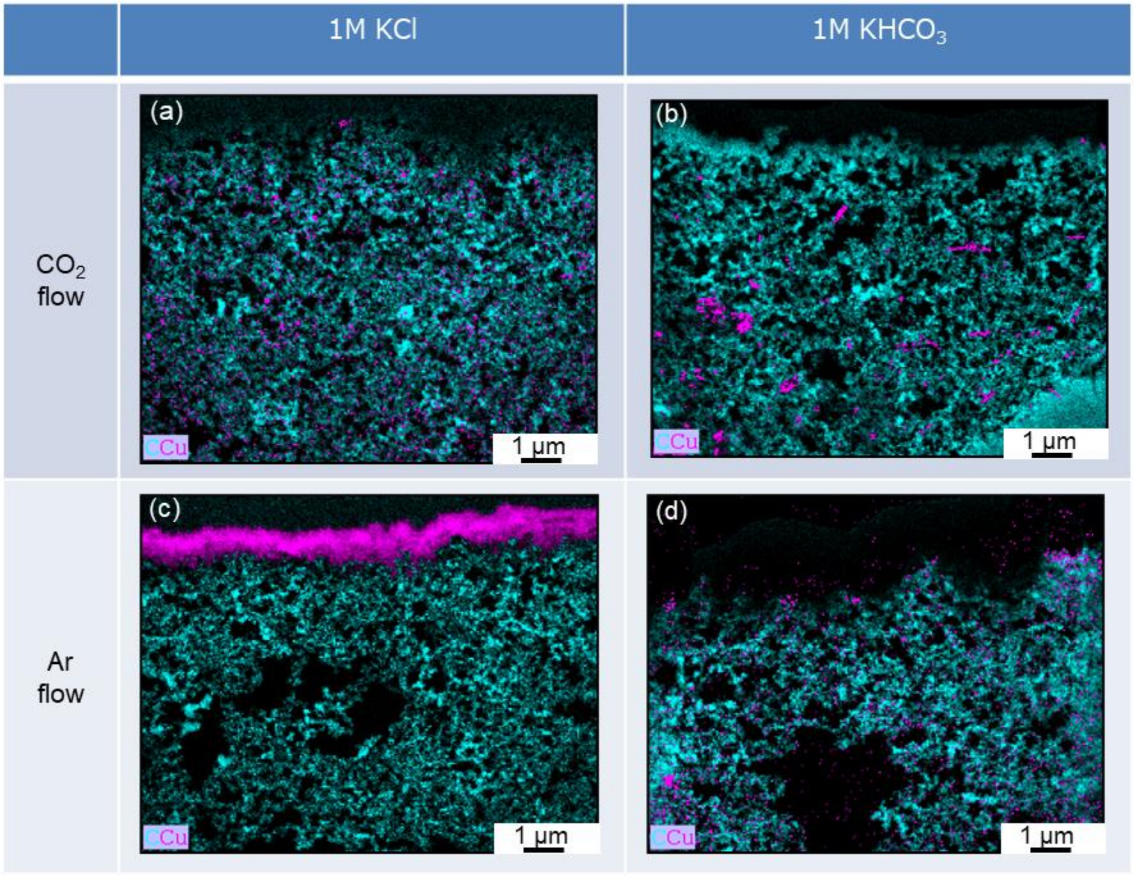

Figure S16 Cross-sectional STEM-EDX mapping of Cu (Cu-K) and C (C-K) elements of Cu(300)-GDE following CP at -400 mA/cm^2 for 20 min: (a) in 1M KCl under CO_2 supply, (b) in 1M $KHCO_3$ under CO_2 supply, (c) 1M KCl under Ar supply, (d) 1M $KHCO_3$ under Ar supply, respectively.

Ex situ STEM-EDX images were obtained using a Hitachi Cs-corrected STEM HD-2700. EDX elemental mappings were obtained utilizing an AMETEK EDAX Octane T Ultra W 100 mm^2 SDD.

Table S1 ICP-MS analysis of the catholyte (1M KCl and 1M KOH) after various conditions under CO_2 or Ar supply. If all the Cu in the pristine Cu(300)-GDE dissolves, the Cu concentration will be ca. 676 µg/L.

Catholyte	Gas supply	Conditions (CP: -400 mA/cm^2)	Cu concentration in catholyte	Dissolved Cu from Cu-GDE
1M KCl	CO_2	OCP 30min	100 µg/L	15 %
		(CP:10 min + OCP:15min) x 3	81 µg/L	12 %
	Ar	OCP 30min	110 µg/L	16 %
		(CP:10 min + OCP:15min) x 3	17 µg/L	3 %
1M KOH	CO_2	OCP 30min	360 µg/L	53 %
		(CP:10 min + OCP:15min) x 3	78 µg/L	12 %
	Ar	OCP 30min	350 µg/L	52 %
		(CP:10 min + OCP:15min) x 3	260 µg/L	38 %

The electrolytes were diluted with dilute nitric acid. The resulting solution was analyzed by inductively coupled plasma mass spectrometry (ICP-MS, Agilent 8800). The standard solutions were prepared using reference materials manufactured by CERI, a designated calibration institute under the JCSS (Japan Calibration Service System), according to the procedure manual. The standard was prepared by transferring a precise volume of the solution of interest from its original concentration to a solution containing the measuring solution. Since the sample was diluted with dilute nitric acid, the standard solution was also based on dilute nitric acid at the same concentration.

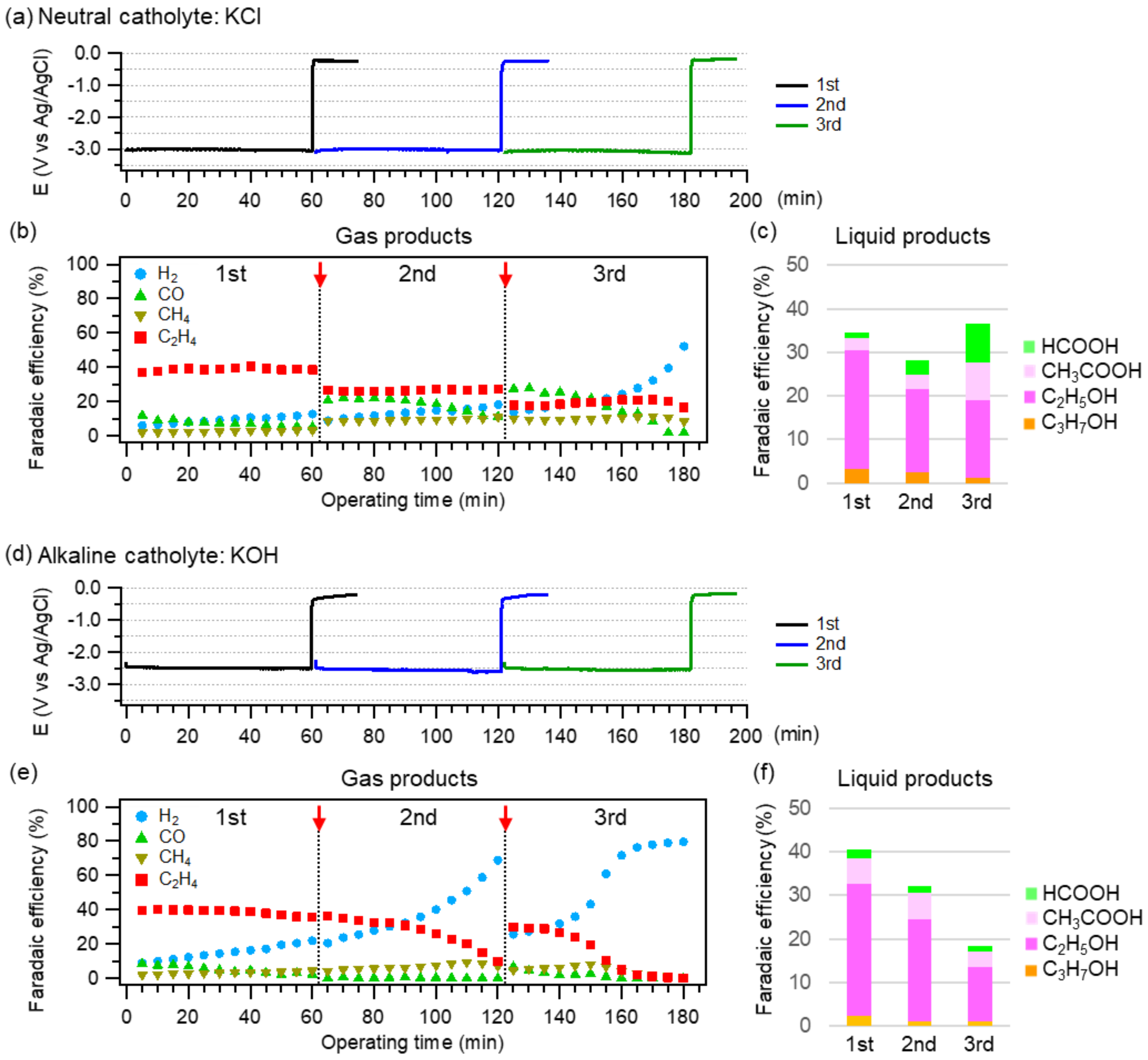

Figure S17 Results of FE evaluation as a function of electrolysis time at -400 mA/cm^2 for 60 min and OCP for 15 min under CO_2 supply. A Cu(300)-GDE was used as cathode, with (a)-(c) KCl catholyte and (d)-(f) KOH catholyte. (a)(d) Potential (V vs Ag/AgCl) without iR compensation and (b)(e) FE curves of gas products (H_2, CO, CH_4, and C_2H_4) as a function of electrolysis time. (c)(f) FEs of liquid products in the recovered catholyte. The washing of the cathode was examined at the position indicated by the red arrow, where the GDE was removed, solvent-washed, and dried, before being reused to reassemble the cell with flesh electrolyte.

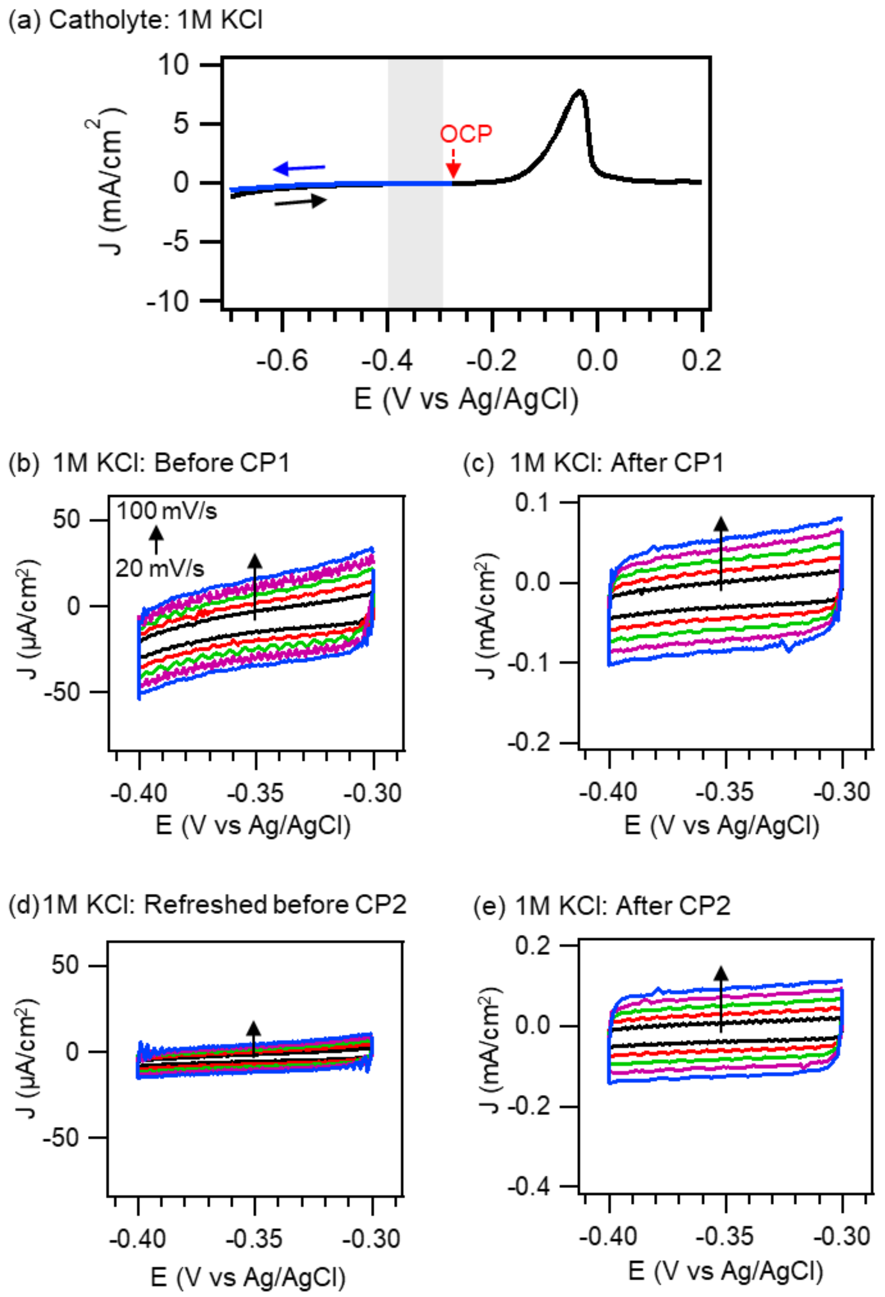

Figure S18 (a) CV obtained at scan rate 2 mV/s and scan range of OCP → -1.0 V → + 0.2V (vs Ag/AgCl) under CO_2 supply in neutral catholyte (1M KCl). The OCP in KCl was ca. -0.28 V (vs Ag/AgCl). The ECSA measurements were conducted in the non-Faraday current region (shaded in gray). (b)-(e) CVs measured in a non-Faradaic region at multiple scan rates: 20, 40, 60, 80 and 100 mV/s for (b) before CP1, (c) after CP1 (-400 mA/cm^2 for 10 min), (d) after washing (refreshed) before CP2, (e) After CP2 (-400 mA/cm^2 for 10 min). The capacitive currents were measured at -0.35 V (vs Ag/AgCl).

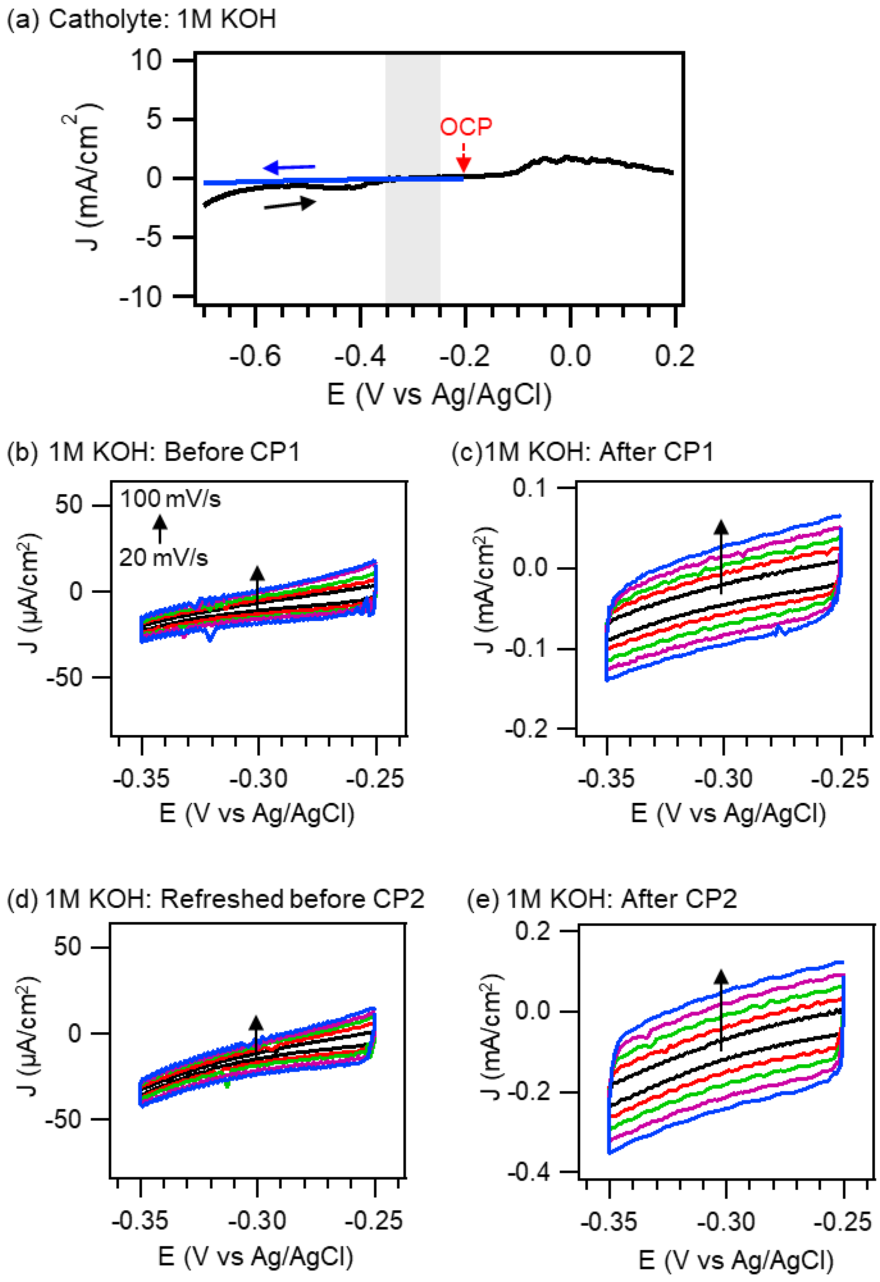

Figure S19 (a) CV obtained at scan rate 2 mV/s and scan range of OCP → -1.0 V → + 0.2V (vs Ag/AgCl) under CO_2 supply in alkaline catholyte (1M KOH). The OCP in KOH was ca. -0.20 V (vs Ag/AgCl). The ECSA measurements were conducted in the non-Faraday current region (shaded in gray). (b)-(e) CVs measured in a non-Faradaic region at multiple scan rates: 20, 40, 60, 80 and 100 mV/s for (b) before CP1, (c) after CP1 (-400 mA/cm^2 for 10 min), (d) after washing (refreshed) before CP2, (e) After CP2 (-400 mA/cm^2 for 10 min). The capacitive currents were measured at -0.30 V (vs Ag/AgCl).

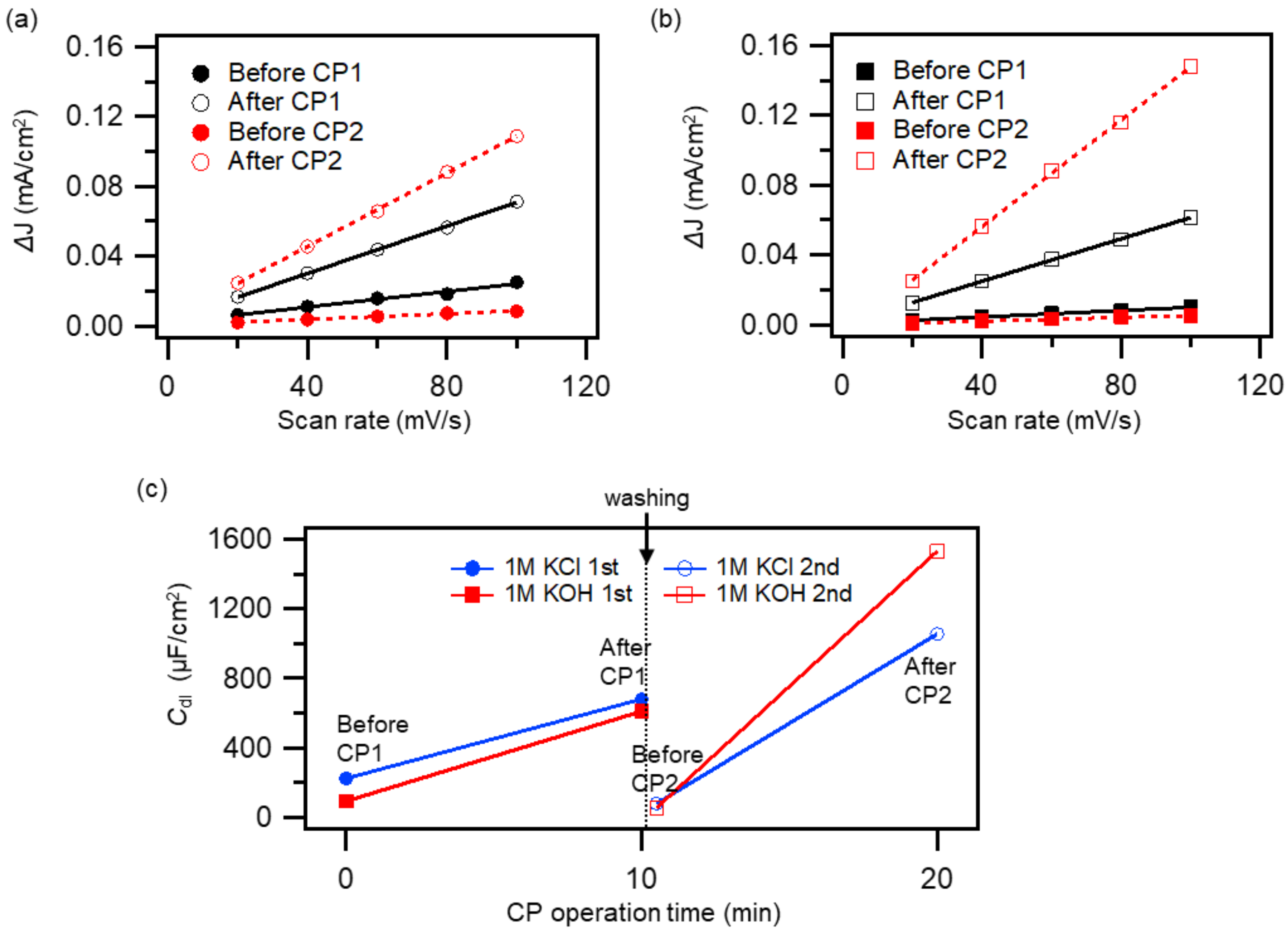

Figure S20 (a) Measured capacitive currents (*ΔJ*) plotted as a function of the scan rate in (a) 1M KCl and (b) 1M KOH. (c) Evolution of double layer charging capacitance (C_{dl}) during electrolysis time, calculated from (a) and (b).

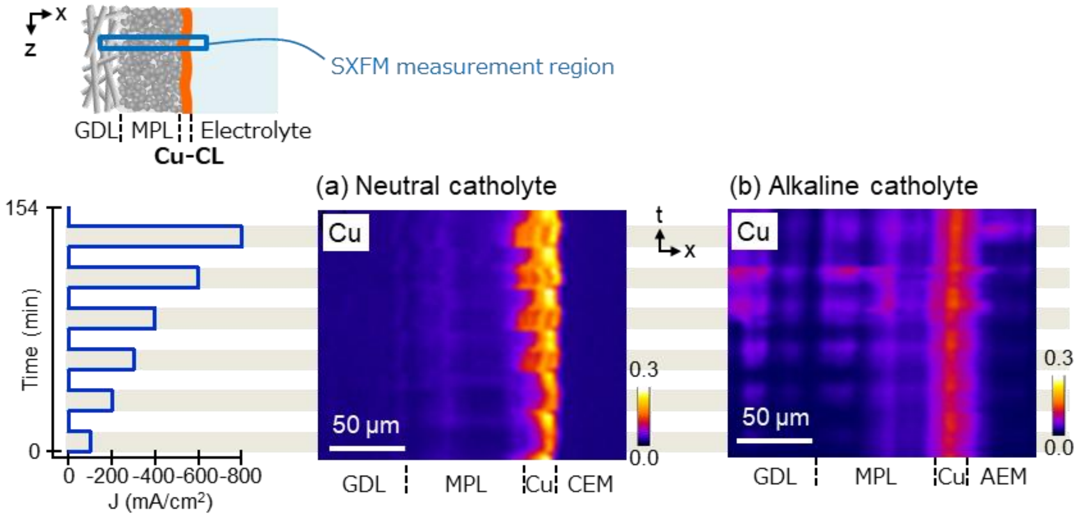

Figure S21 *In situ* SXFM results obtained at SAGA-LS BL07. The Cu maps captured during CP-OCP cycles (current densities: -100, -200, -300, -400, -600, and -800 mA/cm^2) under CO_2 supply, shown as time-dependent Cu intensity profiles along with the x-axis (integrated over the z-axis). (a) In neutral electrolyte (0.5M $KHCO_3$), (b) In alkaline electrolyte (1M KOH).

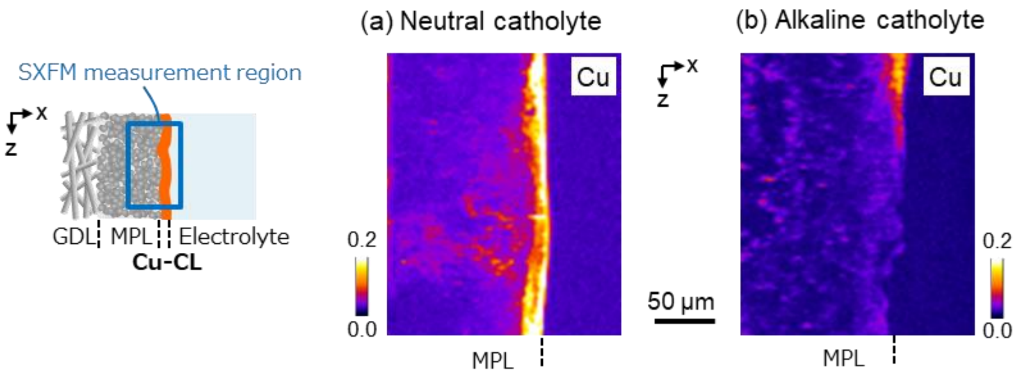

Figure S22 *In situ* SXFM results obtained at SAGA-LS BL07. The Cu map after CP-OCP cycles under CO_2 supply (Figure S21), (a) in neutral electrolyte (0.5M $KHCO_3$) and (b) in alkaline electrolyte (1M KOH).
